# *Kibra* knockdown inhibits the aberrant Hippo pathway, suppresses renal cyst formation and ameliorates renal fibrosis in *nphp1* ^KO^ mice

**DOI:** 10.1002/ctm2.70245

**Published:** 2025-02-24

**Authors:** Yichen Yang, Zhihe Xue, Jiayong Lai, Jinglan Zhang, Changmiao Pang, Jinglin Zhong, Zhanpeng Kuang, Baojuan Zou, Yaqing Liu, Liangzhong Sun

**Affiliations:** ^1^ Department of Pediatrics Nanfang Hospital, Southern Medical University Guangzhou China; ^2^ Department of Pediatrics Shenzhen Maternity and Child Healthcare Hospital Shenzhen China; ^3^ Department of Pediatrics The First Affiliated Hospital, Gannan Medical University Ganzhou China

**Keywords:** Hippo pathway, Kibra, nephronophthisis, nphp1, renal cyst

## Abstract

**Introduction:**

Nephronophthisis (NPH) is an autosomal recessive interstitial cystic kidney disease, which is the most common genetic cause of end‐stage renal disease (ESRD) in childhood. The Hippo pathway is regulated by the cilium and has been suggested to be linked to NPH. The aim of the study was to investigate the involvement of Hippo pathway in the pathogenesis of *nphp1* defect‐associated NPH (NPH1).

**Method:**

*Nphp1* knockout (*nphp1* ^KO^) Madin‐Darby Canine Kidney (MDCK) cells and *nphp1* ^KO^ C57BL/6J mice were generated via CRISPR gene editing strategy. The siRNAs targeting *Kibra*, *MST1* and *LATS1* were designed. An AAV9 vector was designed for *Kibra* knockdown. The expression and phosphorylation of core Hippo pathway molecules were evaluated. Pathological renal changes were evaluated via light microscopy respectively with haematoxylin–eosin and Masson staining.

**Results:**

In *nphp1* ^KO^ MDCK cells, *nphp1* ^KO^ mice and NPH1 patients’ kidneys, Kibra, p‐MST1/2, p‐LATS and p‐YAP exhibited a notable increase in levels, with an even greater elevation observed in renal cyst cells, indicating the Hippo pathway activated in these *nphp1*‐deficient contexts. *Nphp1* re‐expression reversed the Hippo pathway activation in cells, indicating that the Hippo pathway activation is related to *nphp1* deficiency in vitro. Meanwhile, in vitro, *MST1* knockdown downregulated LATS1 and YAP phosphorylation, *LATS1* knockdown downregulated YAP phosphorylation, suggesting the activation of the canonical Hippo pathway in *nphp1*‐deficient contexts. Knockdown of the upstream regulator *Kibra* inhibited the Hippo pathway activation in both *nphp1* ^KO^ MDCK cells and mice. Following *Kibra* knockdown, the organisation of *nphp1* ^KO^ MDCK cells became more compact, the intensity of the actin fibres increased. Besides, decreased renal fibrosis and cyst formation were observed in *nphp1* ^KO^ mice.

**Conclusions:**

The canonical Hippo pathway is aberrantly activated in *nphp1*‐deficient conditions. *Kibra* may serve as a crucial upstream regulator of *nphp1* deficiency‐related Hippo pathway activation. *Kibra* upregulation and activation of the Hippo pathway are involved in the pathogenesis of NPH1.

**Key Points:**

- Canonical Hippo pathway activated in *nphp1*‐deficient disease models and patients.
- Kibra was a key upstream molecule in regulating the activation of canonical Hippo pathway in *nphp1*‐deficient disease models and patients and closely related to renal cyst formation and fibrosis in *nphp1* ^KO^ mice.

## Graphical abstract

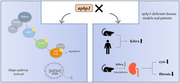

## 1 INTRODUCTION

Nephronophthisis (NPH) is an autosomal recessive interstitial cystic kidney disease that accounts for 10%–20% of the end‐stage renal disease (ESRD) cases in children and adolescents.^1^ Over 20 causative genes have been identified, among which *nphp1* is predominant. *Nphp1* mutation causes type 1 NPH (NPH1).^2^, ^3^, ^4^ Typical pathological changes in patients with NPH include corticomedullary tubular atrophy, dilatation, cyst formation, tubular basement membrane thickening and layering, and diffuse tubulointerstitial fibrosis with inflammatory cell infiltration.^5^, ^6^ The pathogenesis of NPH is unclear and no effective therapy has been developed for its clinical management. The proteins encoded by pathogenic genes associated with NPH are structural components of primary cilia and/or are associated with ciliary function.^7^ Primary cilium is antenna‐like sensory organelles that protrude from the surface of most vertebrate cells.^8^ The primary cilium extends from the basal body as a solitary unit, detects extracellular changes and transduces signals intracellularly via different signalling pathways: Hippo, Wnt and TGF‐β pathways,^9^ to regulate cellular activity, development and homeostasis. Ciliary defects caused by genetic defects cause a group of diseases known as ciliopathies, including autosomal dominant/recessive polycystic kidney disease (ADPKD/ARPKD).

The Hippo pathway, which was identified approximately 30 years ago during tissue growth screening in *Drosophila melanogaster*, is an evolutionarily conserved signalling network and is considered as a key player in a number of cellular functions, including cell proliferation, apoptosis, differentiation, organ size regulation, tumourigenesis.^10^, ^11^, ^12^, ^13^ The Hippo pathway comprises three groups of components: upstream regulators, core molecule proteins and downstream effectors. In mammals, the core components include mammalian sterile 20‐like kinases 1 and 2 (MST1/2), salvador homolog 1 (SAV1), large tumour suppressor 1 and 2 (LATS1/2) and Mps one Binder (MOB) kinase activator 1A and 1B (MOB1A/1B). Yes‐associated protein (YAP) and transcriptional coactivator with PSD‐95/discs large/ZO‐1 (PDZ) ‐binding motif (TAZ) are the main downstream effectors. When the Hippo pathway is activated, MST1/2 interact with SAV1 and then phosphorylate SAV1, MOB1 and LATS1/2.^14^, ^15^, ^16^ LATS1/2 then phosphorylate YAP and TAZ directly, phosphorylated YAP and TAZ bind to cytoplasmic 14‐3‐3 proteins and are then retained in the cytoplasm or degraded by ubiquitylation.^17^, ^18^, ^19^, ^20^ When the Hippo pathway is inactivated, dephosphorylated YAP and TAZ translocate into the nucleus and bind to TFs, mainly TEA domain transcription factor 1–4 (TEAD1‐4), to promote or, infrequently, inhibit target gene transcription.^21^ Proteins involved in cell polarity and cell adhesion are upstream regulators of the Hippo pathway. Among these proteins, the most important are kidney and brain expressed protein (Kibra; also known as WWC1), Merlin (Mer; NF2 in mammals) and Expanded (Ex; FRMD6 in mammals). Kibra is a scaffolding protein with two WW domains, a C2‐like domain and a glutamic acid‐rich C‐terminus. It is predominantly expressed in the kidney and brain tissues and acts with NF2 and FRMD6 as an upstream regulator of the Hippo pathway.^22^, ^23^ Kibra can directly bind to LATS1/2 and SAV1, bringing LATS1/2 in proximity to the MST1/2‐SAV1 complex and promoting the phosphorylation of LATS1/2 by MST1/2.^24^

*YAP* deletion is lethal.^25^ Conditional knockout of *YAP* in the nephrogenic lineage causes severe nephrogenesis defects.^26^ *TAZ* deletion causes cystic kidney disease in mice and renal cysts in zebrafish. Renal cyst formation at the corticomedullary junction mimics NPH. A panel of pathogenic genes associated with ciliopathies, such as *Dctn5*, *Kif3a*, *Pkhd1*, *Tsc1* and *Ofd1*, were downregulated upon *TAZ* deletion.^27^, ^28^ Previous studies have demonstrated that mutations in *nphp4*,^29^ *nphp9/Nek8*,^30^ *nphp3* ^31^ and *nphp16/Anks6* ^32^ can modify the activity of the Hippo pathway through direct or indirect interactions with YAP or TAZ. However, the role of the Hippo pathway in the pathogenesis of NPH remains largely unexplored.

In this study, the association of the Hippo pathway with NPH1 was explored using a *nphp1* knockout (*nphp1* ^KO^) mouse model, *nphp1* ^KO^ MDCK cells and renal biopsy specimens from NPH1 patients. We found that the canonical Hippo pathway was aberrantly activated, with sequential activation of the core molecules MST1/2 and LATS1/2, and the effectors YAP/TAZ. The upstream regulator Kibra was found to be significantly upregulated. *Kibra* knockdown partially reversed the activation of the Hippo pathway in *nphp1*‐deficient models both in vitro and in vivo, suppressed renal cyst formation and ameliorated renal fibrosis in *nphp1* ^KO^ mice.

## 2 MATERIALS AND METHODS

### 2.1 Cell culture

The wild‐type (WT) Madin‐Darby Canine Kidney (MDCK) cell line was kindly provided by Professor Xueqing Yu (Kidney Institute, Sun Yat‐sen University, China). The *nphp1*‐knockout (*nphp1* ^KO^) MDCK cell line was generated previously by our group using CRISPR‐Cas9 gene editing, firstly.^33^, ^34^ To get a more stable cell line, we ultimately utilised the *nphp1* ^KO^ MDCK cell line constructed by Ubigene Biosciences through CRISPR gene editing strategy. Briefly, the knockout was achieved by targeting exons 3–6 of the *nphp1* gene. Plasmids expressing Cas9 protein and gRNAs were introduced into MDCK cells via electroporation. Single‐cell clones were selected and validated through polymerase chain reaction (PCR) and genomic sequencing. All the cells were cultured in minimal essential medium (Gibco) supplemented with 10% foetal bovine serum (FBS; Gibco) and 1% sodium pyruvate (Gibco).

### 2.2 Mice

*nphp1* ^KO^ C57BL/6J mice were generated by our group via deletion of exons 2–20 of *nphp1* using CRISPR‐Cas9 gene editing.^35^, ^36^ (AAV9 vectors carrying either recombinant Kibra‐cDNA plasmids or control plasmids were generated by GeneChem Biotechnology. These vectors were administered via tail vein injection to 4‐week‐old mice, with each mouse receiving 8 × 10^10^ copies. Each experimental group consisted of six mice. All mice were euthanised at 12 weeks of age using sodium pentobarbital anaesthesia. All animal experiments were performed according to the Animal Research: Reporting of In Vivo Experiments (ARRIVE 1) guidelines and the protocols were approved by the Institutional Animal Care and Use Committee of Nanfang Hospital, Southern Medical University (Application Number: NFYY‐2020‐0736).

### 2.3 Patient renal tissue

Renal tissue samples from three NPH1 patients and three control patients were obtained via renal biopsy. The three NPH1 patients were 13, 13 and 10 years old, respectively. All harboured the same mutation: homozygous large deletion of *nphp1*. Three control patients were ailing with primary nephrotic syndrome: Control patient 1 was a 14‐year‐old male with minimal change kidney disease. Control patient 2 was an 11‐year‐old male also with minimal change kidney disease. Control patient 3 was a 10‐year‐old female with focal segmental glomerulosclerosis (FSGS). This study was reviewed and approved by the Ethics Committee of Nanfang Hospital (NFEC‐2019‐047) of Southern Medical University.

### 2.4 Transfection

*LATS1*, *Kibra*, *MST1* and negative control small interfering RNAs (siRNAs) were constructed by HANYI. The siRNA sequences used were as follows: *LATS1* siRNA, 5′‐GAUAAAGACACUAGGAAUATT‐3′; *Kibra* siRNA, 5′‐ CCCGGAAGCGGUUGGAGAATT‐3′; and *MST1* siRNA, 5′‐ CAGAAGUGAUUCAGGAAAUTT‐3′. pcDNA3.1‐*nphp1* was synthesised by GenePharma. GP‐transfect‐Mate transfection reagent (GenePharma) was used for transfection, as instructed by the manufacturer. Six hours after transfection, the FBS‐free medium was replaced with medium containing 10% FBS. Subsequently, the cells were cultured for 24 h before RNA extraction or for 48 h before protein extraction.

### 2.5 RNA extraction and real‐time quantitative PCR (qPCR)

Total RNA was extracted using RNA Isolator Total RNA Extraction Reagent (Vazyme) and reversed using HiScript II Q RT SuperMix (Vazyme) according to the manufacturer's instructions. ChamQ SYBR qPCR Master Mix (Low ROX Premixed; Vazyme) was mixed with the primers and cDNA, and a QuantStudio™ 5 Real‐Time PCR System (Thermo Fisher) was used to perform qPCR. The relative mRNA expression levels were calculated using the 2^−ΔCt^ method. The primer sequences are shown in Supporting Information Table 1.

### 2.6 Protein extraction and western blotting

Cells and tissues were lysed via Radio Immunoprecipitation Assay Lysis buffer (RIPA Lysis buffer; Beyotime) supplemented with 1% Phenylmethylsufonyl fluoride (PMSF; Fudebio) and 1% phosphatase inhibitor (8Solarbio). The lysates were clarified by sonication and centrifugation. Subsequently, the diluted proteins were heated at 100°C for 10 min. Western blotting was performed following standard protocols. These blots were incubated with primary antibodies (Supporting Information Table 2) and then with secondary antibodies (Supporting Information Table 2). The blots were visualised with a Tanon scanner. The density of each band was normalised to that of the GAPDH band using Image J software. And three different biological replicates were used for statistical analysis.

### 2.7 Immunohistochemistry (IHC), haematoxylin–eosin (H&E) staining and Masson's trichrome (Masson) staining

Paraffin‐embedded kidney sections were sliced into a 4 µm thickness and used after dewaxing and rehydration. For IHC, antigen retrieval was performed in target retrieval solution using microwave oven. The activity of endogenous peroxidase was blocked using 3% H_2_O_2_. The sections were then blocked with a 5% bovine serum albumin (BSA) solution for 1 h, followed by incubation with primary antibodies (Supporting Information Table 2) at 4°C overnight. A universal two‐step assay kit (PV‐9000; ZSGB‐BIO) and a DAB kit (ZLI‐9018; ZSGB‐BIO) were used for staining. An upright microscope (ECLIPSE Ci‐L plus, Nikon) or an SQS40P Slide Scan System (Shengqiang Technology) was used to examine the sections. For H&E and Masson staining, eosin (DH0055; Leagene) and a Masson's Trichrome Stain Kit (G1340; Solarbio), respectively, were used following manufacturer's protocol. All images were analysed by the ImageJ software.

### 2.8 Statistical analysis

Data are presented as the mean ± standard error of the mean (SEM). A *p* value <.05 was considered statistically significant. Data were analysed using GraphPad Prism version 8.0.2.

## 3 RESULTS

### 3.1 *Nphp1* deficiency activates the canonical Hippo pathway in MDCK cells

**FIGURE 1:**
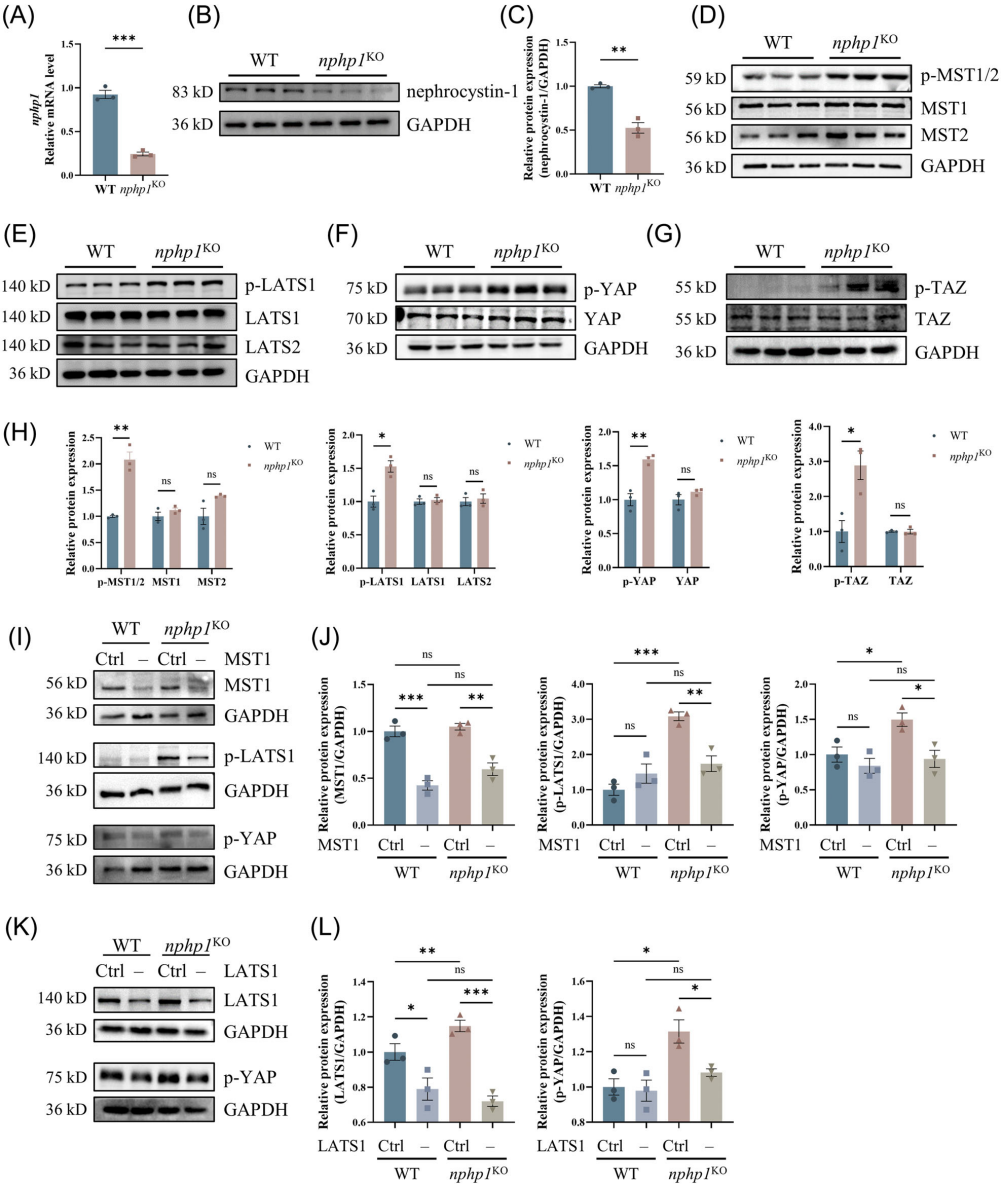
Activation of the canonical Hippo pathway in *nphp1* ^KO^ MDCK cells. (A) The mRNA level of *nphp1* in wild‐type (WT) and *nphp1* ^KO^ MDCK cells was measured by quantitative polymerase chain reaction (qPCR). (B, C) The expression of nephrocystin‐1 in WT and *nphp1* ^KO^ MDCK cells was detected by western blotting (B) and semi‐quantified by densitometric analysis (C). (D–H) The expression of phosphorylation of MST1, MST2, LATS1, LATS2, YAP and TAZ in *nphp1* ^KO^ MDCK cells were detected by western blotting (D–G) and semi‐quantified by densitometric analysis (H). (I, J) The presence of MST1, p‐LATS1 and p‐YAP after *MST1* knockdown was detected by western blotting (I), and their levels were semi‐quantified by densitometric analysis (J). (K, L) The presence of LATS1 and p‐YAP after *LATS1* knockdown was detected by western blotting (K), and their levels were semi‐quantified by densitometric analysis (L). (*n* = 3 biological replicates. The data are presented as the means ± standard error of the means [SEMs]. ∗*p* < .05; ∗∗*p* < .01; ∗∗∗*p* < .001; ns, not significant. *t*‐test or one‐way analysis of variance [ANOVA].)

To explore the status of the Hippo pathway in the context of NPH1, *nphp1* ^KO^ MDCK cells were generated by deleting exons 3–6 of *nphp1* using CRISPR‐Cas9 gene editing. qPCR and western blot results demonstrated a significant reduction in nephrocystin‐1 expression in *nphp1* ^KO^ MDCK cells (Figure 1A–C). The total expression levels of the key components of the Hippo pathway were measured. The protein levels of these key components remained unchanged (Figure 1D–H). However, the phosphorylation of MST1/2, LATS1, YAP and TAZ were significantly increased (Figure 1D–H), indicating that the Hippo pathway was activated in *nphp1* ^KO^ MDCK cells.

The Hippo pathway can be activated via canonical and non‐canonical mechanisms. To determine which pathway was dominantly activated in *nphp1* ^KO^ MDCK cells, we generated siRNAs targeting *MST1* and *LATS1* and transfected them into both WT and *nphp1* ^KO^ MDCK cells, respectively (Figure 1M). As shown in Figure 1I,K, the expression of MST1 or LATS1 was successfully knocked down. *MST1* knockdown significantly decreased both p‐LATS1 and p‐YAP levels (Figure 1I,K), while *LATS1* knockdown significantly decreased p‐YAP level in *nphp1* ^KO^ MDCK cells (Figure 1K,L). These results indicated that the Hippo pathway is activated through the canonical mechanism in *nphp1* ^KO^ MDCK cells.

### 3.2 *Nphp1* re‐expression reversed the Hippo pathway activation in *nphp1* ^KO^ MDCK cells

**FIGURE 2:**
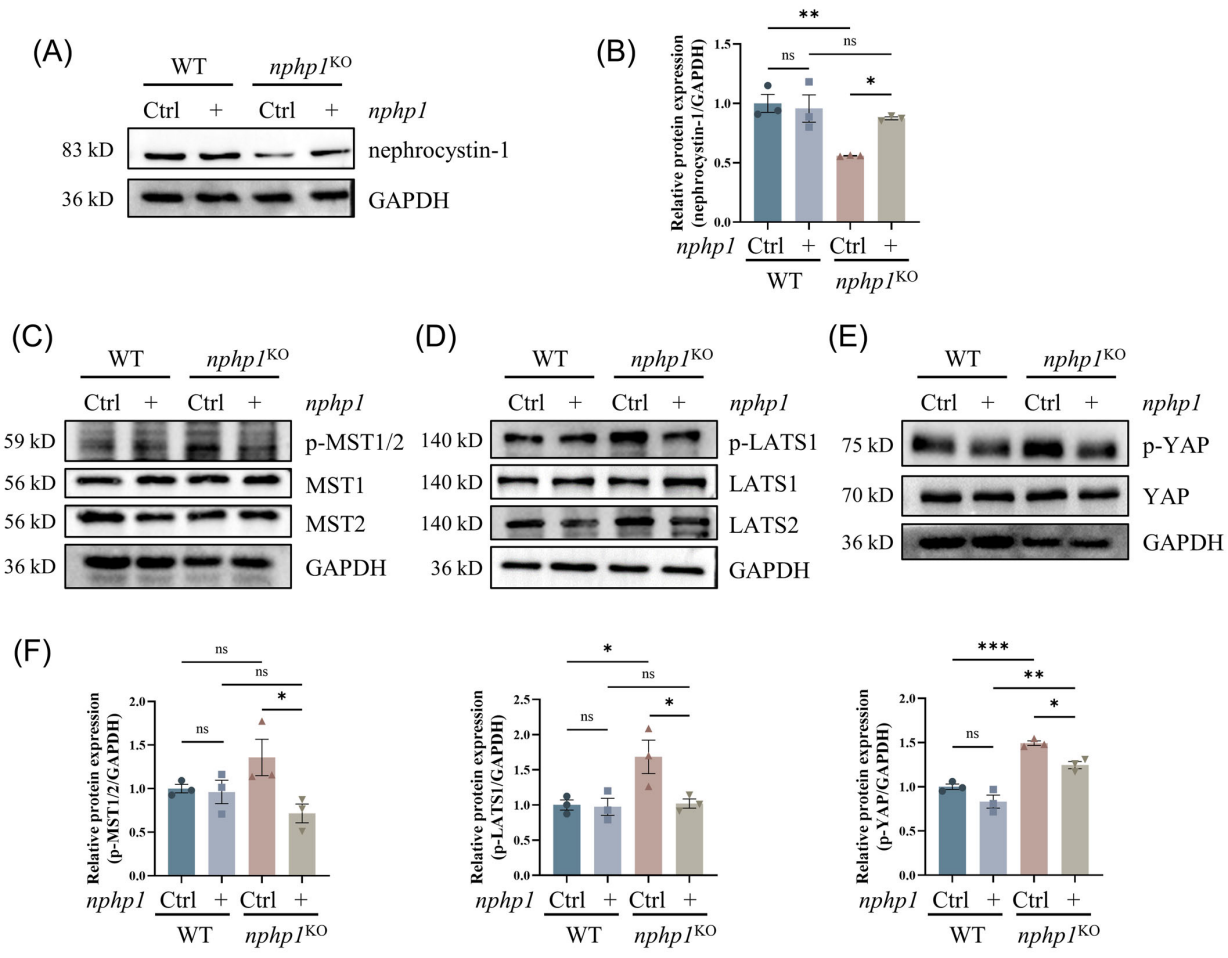
*Nphp1* re‐expression reversed the activation of the Hippo pathway in *nphp1* ^KO^ MDCK cells. (A, B) The expression of nephrocystin‐1 after *nphp1* re‐expression was detected by western blotting (A) and semi‐quantified by densitometric analysis (B). (C–F) The expression of MST1, MST2, LATS1, LATS2, YAP, and the phosphorylation of MST1/2, LATS1 and YAP were detected by western blotting (C–E) and semi‐quantified by densitometric analysis (F). (*n* = 3 biological replicates. All values are means  ±   standard error of the mean [SEM]. ∗*p* < .05; ∗∗*p* < .01; ∗∗∗*p* < .001; ns, not significant. One‐way analysis of variance [ANOVA].)

To verify whether the activation of the Hippo pathway in the NPH1 model resulted from *nphp1* deficiency, we constructed a *nphp1* overexpression lentiviral plasmid and transduced it into *nphp1* ^KO^ MDCK cells. Successful re‐expression of *nphp1* was confirmed by western blotting (Figure 2A,B). Re‐expression of *nphp1* in *nphp1* ^KO^ MDCK cells led to a reversal in the increased levels of p‐MST1/2, p‐LATS1 and p‐YAP, whereas no significant alterations were observed in the levels of the non‐phosphorylated forms of the core Hippo pathway proteins (Figure 2C–F and Supporting Information Figure 1). These findings indicated that *nphp1* re‐expression reversed the abnormal activation of the Hippo pathway in *nphp1* ^KO^ MDCK cells and that the activation of the Hippo pathway was associated with the deficiency of *nphp1* in *nphp1* ^KO^ MDCK cells.

### 3.3 The Hippo pathway is activated in the kidneys of *nphp1* ^KO^ mice and human NPH1 patients

**FIGURE 3:**
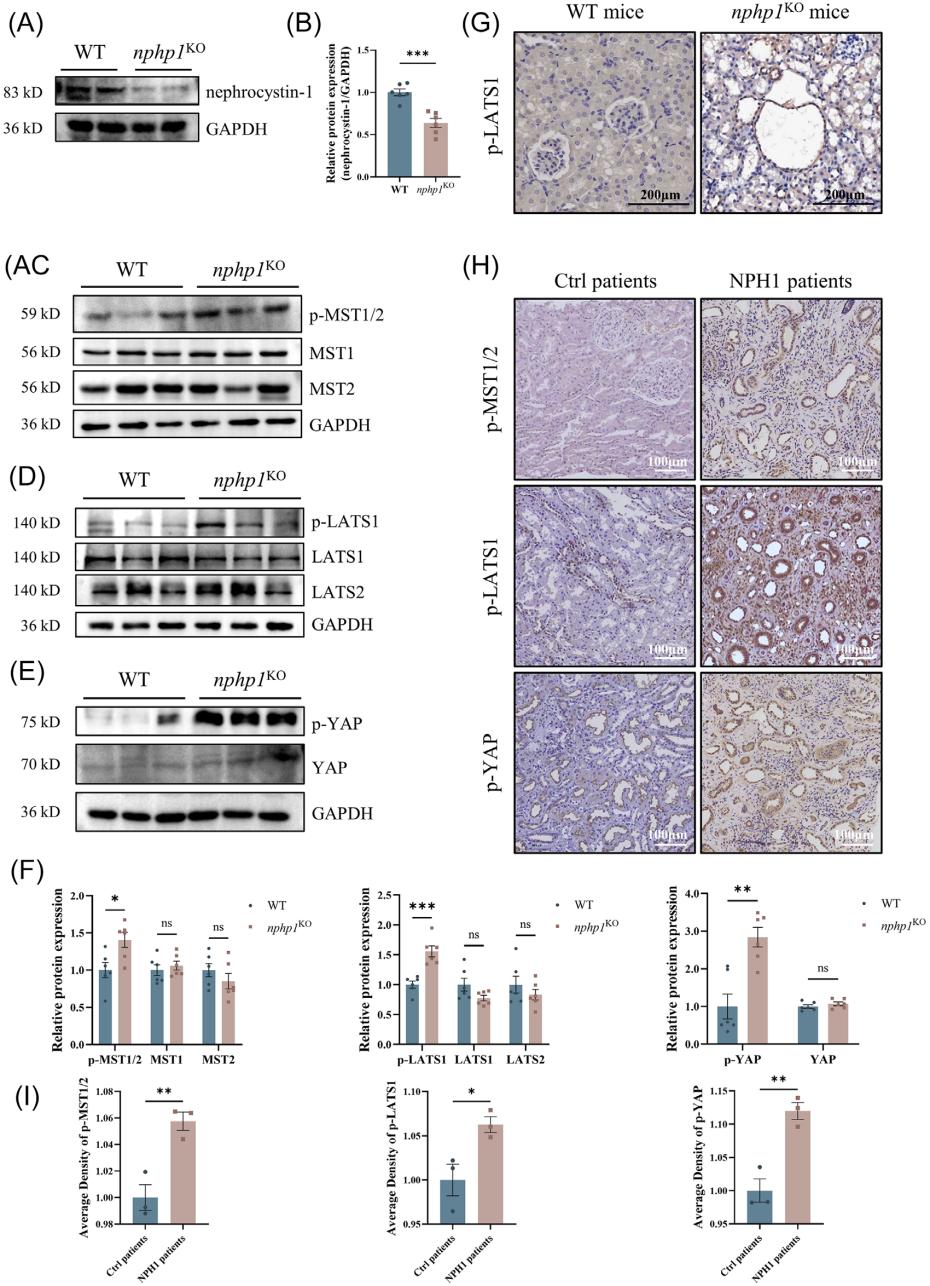
Activation of the Hippo pathway in the kidneys of *nphp1* ^KO^ mice and human NPH1 patients. (A, B) Nephrocystin‐1 in wild‐type (WT) and *nphp1* ^KO^ mice was detected by western blotting (A) and semi‐quantified by densitometric analysis (B). (C–F) The expression of MST1, MST2, LATS1, LATS2 and YAP and the phosphorylation of MST1/2, LATS1 and YAP in the kidneys of WT and *nphp1* ^KO^ mice were detected by western blotting (C–E) and semi‐quantified by densitometric analysis (F). (*n* = 6 mice/group. The data are presented as mean ± standard error of the means [SEMs]. ∗*p* < .05; ∗∗*p* < .01; ∗∗∗*p* < .001; ns, not significant. *t*‐test.) (G) The presence of p‐LATS1 in renal cysts of WT and *nphp1* ^KO^ mice was evaluated using immunohistochemistry (IHC). (Scale bar = 100 µm.) (H, I) The phosphorylation of MST1/2, LATS1 and YAP in renal tissue from nephrotic syndrome patients (Ctrl) and NPH1 patients was evaluated by IHC (H), and their levels were semi‐quantified by average density analysis (I). (*n* = 3 patients/group. Scale bar = 100 µm. The data are presented as mean ± SEMs ∗*p* < .05; ∗∗*p* < .01. *t*‐test.)

To assess if the Hippo pathway is also activated in vivo, we used *nphp1* ^KO^ mice with an NPH renal phenotype, previously generated by our group using CRISPR‐Cas9 gene editing.^36^ The knockout efficiency of nephrocystin‐1 in *nphp1* ^KO^ mice was reconfirmed by western blotting (Figure 3A,B). As expected, p‐MST1/2, p‐LATS1 and p‐YAP levels in renal tissue exhibited an increase in *nphp1* ^KO^ mice, while the levels of non‐phosphorylated LATS1, LATS2, MST1, MST2 and YAP were not significantly changed in *nphp1* ^KO^ mice compared with WT mice (Figure 3C–E). IHC revealed strong positivity for p‐LATS1 in *nphp1* ^KO^ mice's renal cyst cells, but weak positivity in WT mice (Figure 3G).

Furthermore, we selected 3 NPH1 patients and 3 control patients with primary nephrotic syndrome and found that phosphorylated MST1/2, LATS1 and YAP levels in renal tissue were higher in NPH1 patients than in nephrotic syndrome patients (Figure 3H). In the renal cysts of NPH1 patients, the phosphorylation of these molecules was more prominent (Figure 3I).

These results suggested that the Hippo pathway was also activated in the kidneys of both *nphp1* ^KO^ mice and NPH1 patients and was much more strongly activated within renal cyst cells, highlighting its potential role in renal cyst formation in NPH.

### 3.4 *Kibra* knockdown inhibits the activation of the Hippo pathway in *nphp1* ^KO^ MDCK cells

**FIGURE 4:**
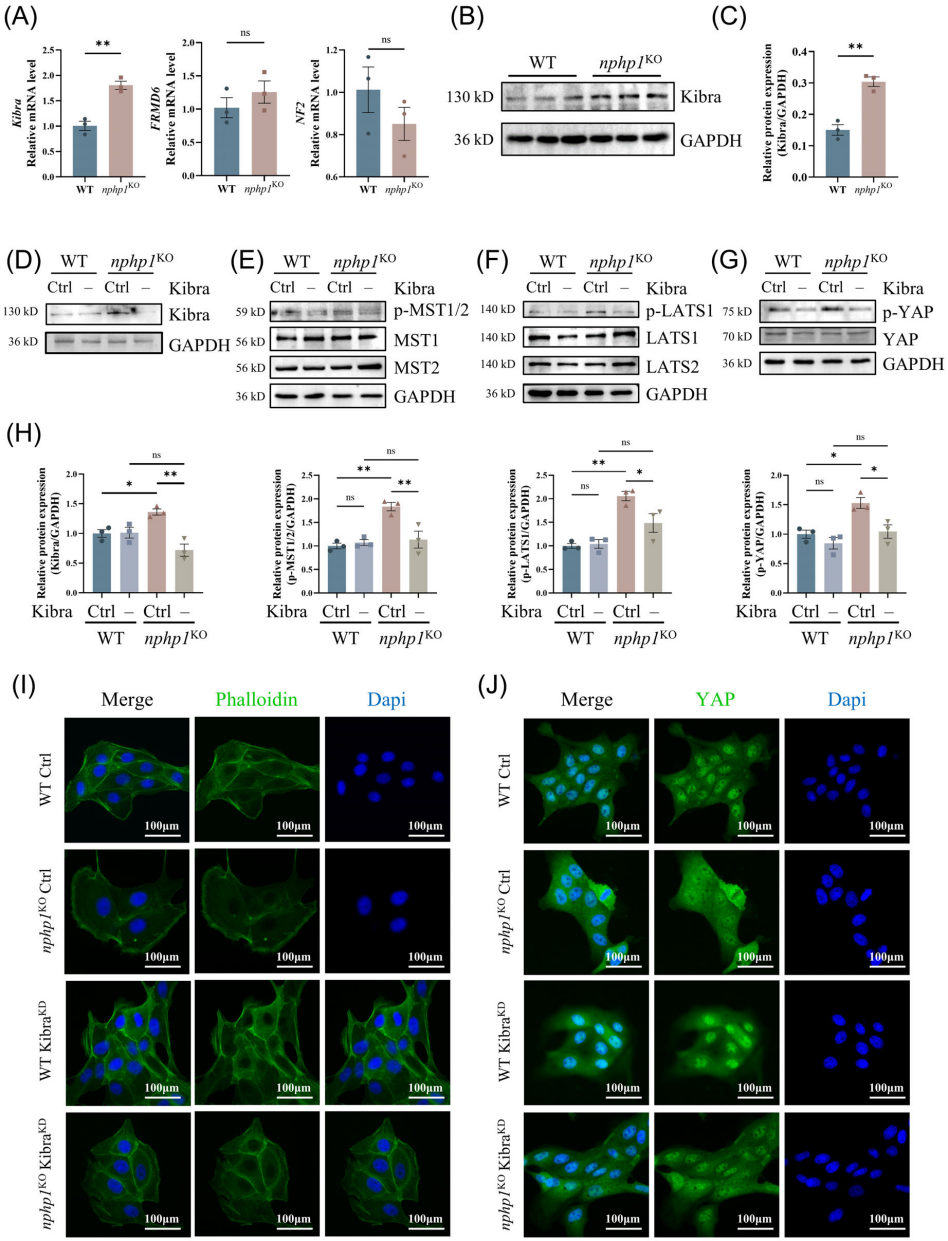
*Kibra* knockdown inhibited the Hippo pathway in *nphp1* ^KO^ MDCK cells. (A) The mRNA levels of *Kibra*, *NF2* and *FRMD6* in wild‐type (WT) and *nphp1* ^KO^ MDCK cells were measured using quantitative polymerase chain reaction (qPCR). (B, C) The expression of Kibra was detected by western blotting (B) and semi‐quantified by densitometric analysis (C). (D–H) The expression of Kibra, MST1, MST2, LATS1, LATS2, YAP, p‐MST1/2, p‐LATS1 and p‐YAP after *Kibra* knockdown were detected by western blotting (D–G) and semi‐quantified by densitometric analysis (H). (*n* = 3 biological replicates. The data are presented as mean ± standard error of the means [SEMs]. ∗*p* < .05; ∗∗*p* < .01; ns, not significant. *t*‐test or one‐way analysis of variance [ANOVA].) (I) Effects of *Kibra* knockdown on cytoskeleton arrangement. (J) Effects of *Kibra* knockdown on YAP nuclear localisation. Scale = 100 µm.

Despite the abovementioned findings, the mechanism through which *nphp1* deficiency induces the activation of the Hippo pathway is not clear. The canonical Hippo pathway activation is related to a set of upstream activation factors such as Kibra, NF2 and FMRD6. Next, we detected the expression of the molecules upstream of the Hippo pathway in *nphp1* ^KO^ MDCK cells. Compared with the expression levels in WT MDCK cells, *Kibra* and *FMRD6* mRNA expression was upregulated in *nphp1* ^KO^ MDCK cells, with *Kibra* upregulation being more significant. The change in *NF2* expression was not statistically significant (Figure 4A). Western blotting analysis confirmed the upregulation of Kibra protein expression (Figure 4B,C).

To explore whether Kibra upregulation mediates the activation of the Hippo pathway in *nphp1* ^KO^ models, we transfected both WT and *nphp1* ^KO^ MDCK cells with *Kibra* knockdown siRNA (si‐*Kibra*) and control siRNA (si‐Ctrl), respectively. The efficiency of *Kibra* knockdown was confirmed by western blotting (Figure 4D,H). Although there were no significant changes in the levels of MST1/2, LATS1/2 and YAP, the phosphorylation of MST1/2, LATS1 and YAP decreased significantly in *nphp1* ^KO^ MDCK cells upon si‐*Kibra* transfection compared to si‐Ctrl transfection (Figure 4E–H). In the WT group, the changes in the levels of both phosphorylated and non‐phosphorylated forms of these proteins were not significant (Figure 4E–H).

Besides, WT MDCK cells were tightly arranged with clear outlines, and their actin fibres were organised around the periphery of the cell. However, in *nphp1* ^KO^ MDCK cells, the arrangement of cells was disturbed with increased volume in a few cells. The actin fibres surrounding these enlarged cells exhibited weaker intensity. Upon knockdown of *Kibra*, the organisation of *nphp1* ^KO^ MDCK cells became more compact, and the intensity of the actin fibres increased (Figure 4I).

As we expected, in WT MDCK cells, YAP was distributed in both cytoplasm and nucleus, with evident nuclear localisation. In *nphp1* ^KO^ MDCK cells, however, YAP was more inclined to be distributed in the cytoplasm. Knocking down *Kibra* promoted nuclear localisation of YAP in *nphp1* ^KO^ MDCK cells (Figure 4J and the statistical analysis is presented in Supporting Information Figure 2).

### 3.5 *Kibra* knockdown inhibits the activation of the Hippo pathway in *nphp1* ^KO^ mice

**FIGURE 5:**
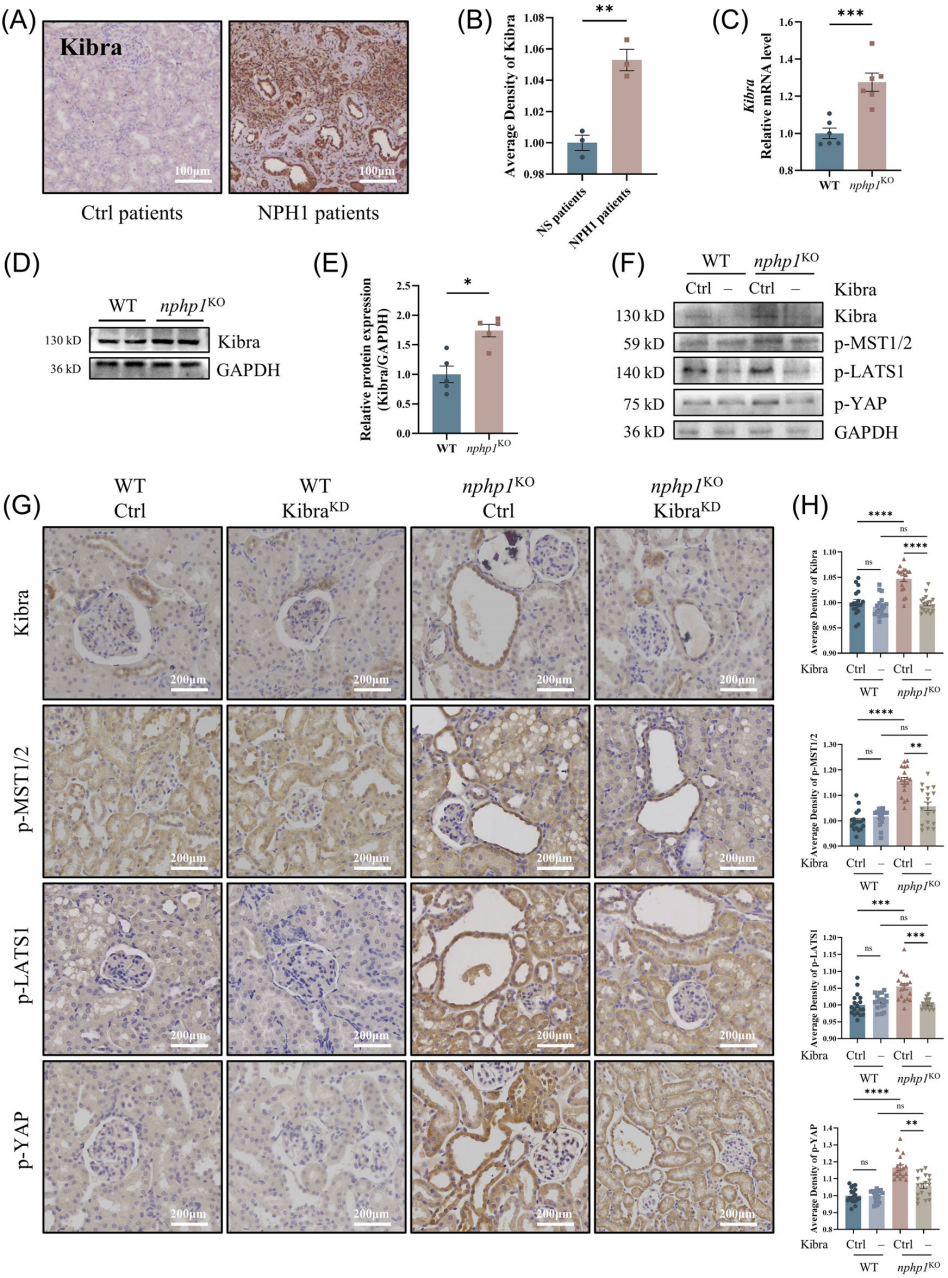
*Kibra* knockdown inhibited the activation of the Hippo pathway in the kidneys of *nphp1* ^KO^ mice. (A, B) The expression of Kibra in the renal tissue of nephrotic syndrome patients (Ctrl) and NPH1 patients was examined by immunohistochemistry (IHC) (A) and semi‐quantified by density analysis (B). (*n* = 3 patients/group. Scale bar = 100 µm. ∗∗*p* < .01. *t*‐test). (C) The mRNA levels of *Kibra* were measured by quantitative polymerase chain reaction (qPCR). (D, E) The expression of Kibra was detected by western blotting (D) and semi‐quantified by densitometric analysis (E). (*n* = 6 or 5 mice/group. The data are presented as mean ± standard error of the means [SEMs]. ∗*p* < .05; ∗∗∗*p* < .001. *t*‐test.) (F) The expression of Kibra, p‐MST1/2, p‐LATS1 and p‐YAP in the kidney of mice were detected by western blotting. (G, H) The presence of Kibra, p‐MST1/2, p‐LATS1 and p‐YAP in mice kidney was examined by IHC (G), and their levels were semi‐quantified by average density analysis (H) (*n* = 6 mice/group, 3 images/mouse. Scale bar = 100 µm. The data are presented as mean ± SEMs. ∗*p* < .05; ∗∗*p* < .01; ∗∗∗*p* < .001; ∗∗∗∗*p* < .0001; ns, not significant. One‐way analysis of variance [ANOVA].)

We further evaluated the expression of Kibra in vivo. Kibra was upregulated in the renal tissues of NPH1 patients (Figure 5A,B) and in the kidneys of *nphp1* ^KO^ mice (Figure 5C–E). To explore the effect of *Kibra* knockdown on the Hippo pathway activation in *nphp1* ^KO^ mice, we designed a *Kibra* knockdown short hairpin RNA (shRNA) construct and inserted it into an AAV9 vector (*Kibra* ^KD^‐AAV9). We introduced *Kibra* ^KD^‐AAV9 into both WT and *nphp1* ^KO^ mice at 4 weeks of age via tail vein injection. The efficiency of *Kibra* knockdown in the kidney tissue was confirmed by western blotting (Figure 5F) and IHC (Figure 5G,H). Upon *Kibra* knockdown, the phosphorylation of MST1/2, LATS1 and YAP decreased in the kidneys of *nphp1* ^KO^ mice, but the differences in the phosphorylation of these proteins between the kidneys of WT mice without or with *Kibra* knockdown were not significant (Figure 5F–H and Supporting Information Figure 3). Moreover, the Kibra, p‐MST1/2, p‐LATS1 and p‐YAP were more positive in or around the renal cysts. These results suggested that *Kibra* knockdown can inhibit the activation of the Hippo pathway in the kidneys of *nphp1* ^KO^ mice.

### 3.6 *Kibra* knockdown suppresses renal cyst formation and ameliorates renal fibrosis in *nphp1* ^KO^ mice

**FIGURE 6:**
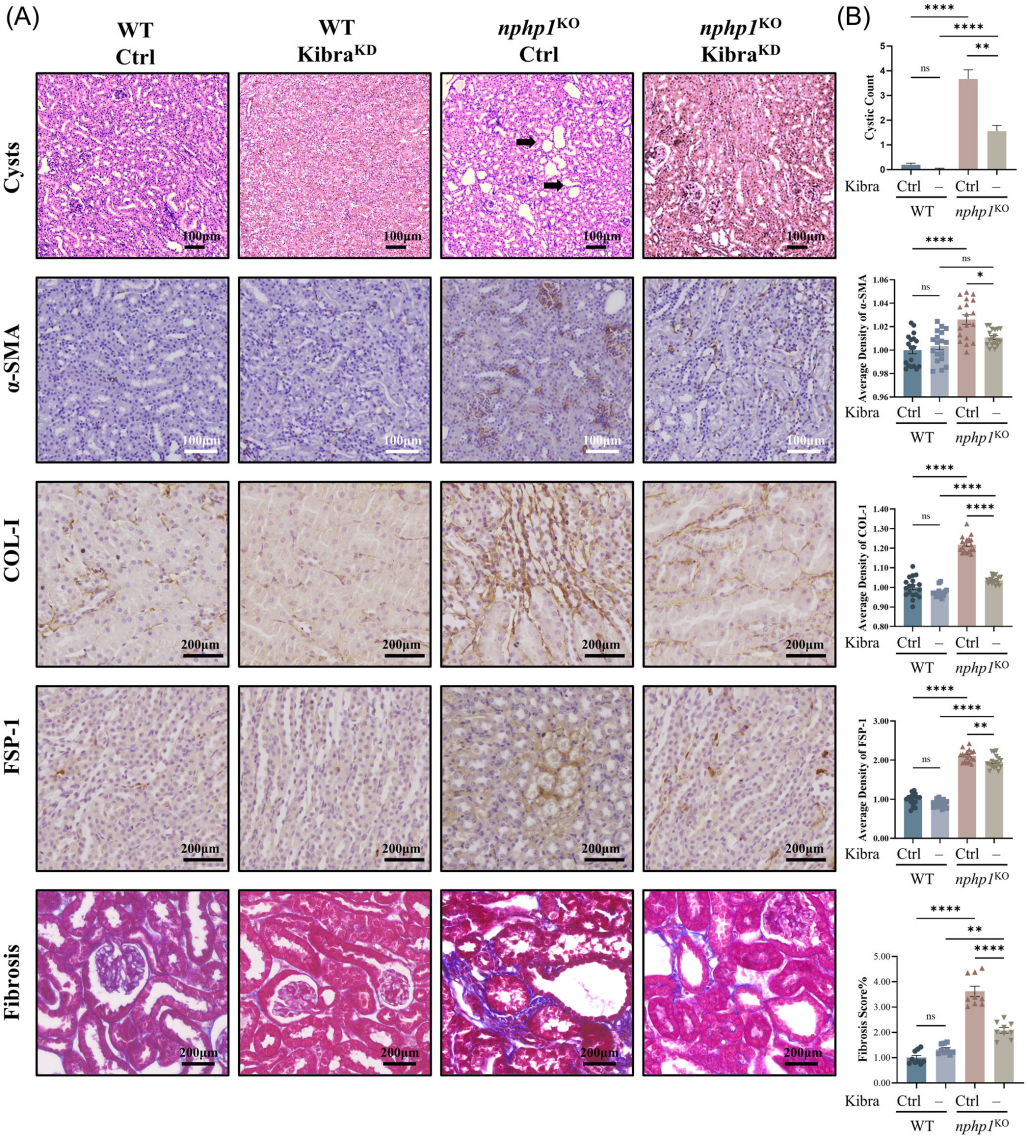
*Kibra* knockdown suppressed renal cyst formation and ameliorates renal fibrosis in *nphp1* ^KO^ mice. (A) Haematoxylin–eosin (H&E) staining of kidney tissue showing renal cysts (black arrows) in *nphp1* ^KO^ mice. The expression of α‐SMA, FSP‐1 and COL‐1 were examined by immunohistochemistry (IHC). Masson staining showed fibrosis in kidney tissue of *nphp1* ^KO^ mice. (B) Semi‐quantitative analysis of the renal cyst count, average density of the expression of α‐SMA, COL‐1, FSP‐1 and fibrosis score (%) in the different groups of mice. (*n* = 6 mice/group, 3 images/mouse. Scale bar = 100 µm or 200 µm. The data are presented as mean ± standard error of the means [SEMs]. ∗*p* < .05; ∗∗*p* < .01; ∗∗∗*p* < .001; ∗∗∗∗*p* < .0001; ns, not significant. One‐way analysis of variance [ANOVA].)

Our pathological analysis revealed a significant decrease in renal cyst formation in *Kibra*‐knockdown *nphp1* ^KO^ mice compared to that in negative control *nphp1* ^KO^ mice (Figure 6A,B). IHC showed that expression of α‐SMA, COL‐1, FSP‐1 was lower in *Kibra*‐knockdown *nphp1* ^KO^ mice than in negative control mice (Figure 6A,B). The renal fibrosis score was evaluated by Masson staining, which also revealed an improvement in renal fibrosis in *Kibra*‐knockdown *nphp1* ^KO^ mice compared to negative control mice (Figure 6A,B). These findings reveal that *Kibra* knockdown can effectively reduce renal cyst formation and ameliorate fibrosis in *nphp1* ^KO^ mice, suggesting that Kibra may serve as a potential therapeutic target for NPH1.

## 4 DISCUSSION

In this study, by using *nphp1* knockout models, we demonstrated that the canonical Hippo pathway was aberrantly activated in the context of *nphp1* deficiency both in vitro and in vivo and was highly activated in *nphp1*‐deficient renal cyst cells. *nphp1* deficiency induced the activation of the canonical Hippo pathway via the sequential phosphorylation of all the core molecules. When *nphp1* was re‐expressed in *nphp1* ^KO^ MDCK cells, the activation of the Hippo pathway was inhibited. Activation of the Hippo pathway has previously been observed in the context of defects in other NPH‐associated pathogenic genes, such as *nphp4*, *nphp9/Nek8*, *nphp3* and *nphp16/Anks6*. Nephrocystin‐4 inhibits phosphorylation of YAP and TAZ via direct interaction with LATS1. This inhibition is removed when *nphp4* is knocked down, leading to the phosphorylation and cytoplasmic sequestration of YAP.^29^ *Nek8* directly interacts with TAZ to form the TAZ/*Nek8* complex, which facilitates the nuclear translocation of TAZ.^30^ Nephrocystin‐3 promotes TAZ/TEAD‐driven transcriptional activity.^31^ *Anks6* mutants have been found to exhibit altered ciliary localisation, promote YAP expression and increase both its nuclear and cytoplasmic accumulation.^37^ We found that the upstream molecule of the Hippo pathway, Kibra, was prominently upregulated in the context of *nphp1* deficiency. *Kibra* knockdown decreased the phosphorylation of the core proteins MST1/2 and LATS1 as well as the phosphorylation of YAP in *nphp1* ^KO^ models. Our results showed that the canonical Hippo pathway was activated in models of *nphp1* deficiency via the sequential activation of core proteins. Furthermore, *Kibra* knockdown reduced renal cyst formation and ameliorated renal fibrosis in *nphp1* ^KO^ mice.

The Hippo pathway controls organ size and regeneration by regulating cell proliferation, differentiation, survival and apoptosis. In ADPDK, inactivation of the Hippo pathway has been evidenced and may facilitate continuous growth of renal cysts until the late stage of the disease, leading to the enlargement of kidneys and the loss of their normal shape.^38^ In contrast, the Hippo pathway is active under most conditions in NPH, as reported previously^29^, ^30^, ^32^ and observed in this study in *nphp1* ^KO^ models and NPH1 patients. Activation of the Hippo pathway in NPH has been proposed to limit cyst growth and contribute to maintaining the normal size of the kidney.^38^ We observed a significant enhancement in the levels of p‐MST1/2, p‐LATS and p‐YAP within renal cyst cells in NPH1 patients, which may indeed limit cyst growth and contribute to maintenance of the kidney size. However, when the Hippo pathway was inhibited by *Kibra* knockdown, the number of renal cysts decreased in the *nphp1* ^KO^ mice, indicating that the mechanism of renal cyst formation in NPH may be far more complex than previously thought and is worthy of further study.

We also observed that Kibra knockdown and Hippo inhibition ameliorated renal fibrosis in *nphp1* ^KO^ mice. In contrast to the situation in ADPKD, in which renal fibrosis develops only at a late stage and may be secondary to renal cyst growth, renal fibrosis in NPH develops at an early stage, even before renal tubular dilatation and/or cyst formation.^39^, ^40^ Therefore, renal fibrosis in NPH is considered to be an intrinsic disorder, although the underlying mechanism is unclear.

TGF‐β/SMAD and Wnt pathways are the two main pathways that mediate renal fibrosis in different contexts of kidney impairment. Both signalling pathways are associated with cilium.^9^ Many studies have shown that Hippo pathway participates in crosstalk with both TGF‐β and Wnt signalling pathways at different levels.^41^ However, these crosstalks are highly context‐specific, and their association with renal fibrosis in NPH still needs to be determined.

In addition to its role as a key upstream regulator of the Hippo pathway in regulating cell proliferation, Kibra could regulate the actin cytoskeleton and focal adhesions either directly or indirectly. Kibra associates with synaptopodin directly to regulate the actin cytoskeleton in podocytes.^42^ Kibra can directly interact with the scaffolding protein angiomotin (Amot) to enhance the assembly of actin filament at focal adhesions in endothelial cells.^43^, ^44^ *Kibra* overexpression disrupts the actin cytoskeleton structure in podocytes.^45^, ^46^ Podocyte‐specific *Kibra* overexpression in vivo predisposed experimental mice to acute and chronic podocyte and glomerular impairment.^46^ Increased Kibra expression was observed in patients' cohorts of FSGS and glomerular kidney disease, where it was found to be associated with glomerular disease progression.^45^, ^46^ Consistent with these findings, we also found that upregulation of Kibra participates in the disarrangement of the actin skeleton in renal tubular cells in the context of *nphp1* deficiency. Kibra expression was increased in renal tubular cells in both the in vivo and in vitro *nphp1* ^KO^ models, as well as in renal biopsy tissue from NPH1 patients. Changes in cell morphology and actin cytoskeleton disarrangement were observed in *nphp1* ^KO^ MDCK cells. Kibra knockdown reversed the alterations in cell morphology and disarrangement of the cytoskeleton in *nphp1* ^KO^ MDCK cells. Whether the disarrangement of the actin cytoskeleton associated with Kibra upregulation contributes to disease progression and renal fibrosis in NPH1 deserves further investigation. These findings indicated that Kibra may be a potential therapeutic target for NPH1.

Overall, our results showed that the canonical Hippo pathway was aberrantly activated both in vivo and in vitro models of *nphp1* deficiency, as well as in the kidneys of human NPH1 patients, and was highly active in renal cyst cells. Activation of the Hippo pathway may contribute to the maintenance of kidney size and the inhibition of renal cyst growth in patients with NPH. Kibra, a key upstream regulator of the Hippo pathway, was significantly upregulated in the context of *nphp1* deficiency. Kibra upregulation was associated with aberrant activation of the Hippo pathway and cytoskeletal disarrangement, as well as with renal cyst formation and renal fibrosis. However, further studies are needed to clarify the mechanism by which the Kibra and the Hippo pathway contribute to the pathogenesis of NPH1.

## AUTHOR CONTRIBUTIONS

Conceptualisation: Liangzhong Sun. Data curation: Yichen Yang, Zhihe Xue, Jiayong Lai. Formal analysis: Yichen Yang, Zhihe Xue, Jiayong Lai. Funding acquisition: Liangzhong Sun, Yaqing Liu. Investigation: Yichen Yang, Zhihe Xue, Jiayong Lai, Jinglan Zhang, Changmiao Pang, Jinglin Zhong, Zhanpeng Kuang, Baojuan Zou. Project administration: Liangzhong Sun. Resources: Liangzhong Sun, Yaqing Liu. Supervision: Liangzhong Sun, Yaqing Liu. Validation: Yichen Yang, Zhihe Xue, Jiayong Lai, Jinglan Zhang. Visualisation: Yichen Yang, Zhihe Xue, Jiayong Lai. Writing—original draft: Yichen Yang, Zhihe Xue, Jiayong Lai. Writing – review & editing: Liangzhong Sun, Yaqing Liu.

## CONFLICT OF INTEREST STATEMENT

The authors declare no conflicts of interest.

### ETHICES STATEMENT

The use of clinical samples for the present study was approved by the Ethics Committee of Nanfang Hospital (NFEC‐2019‐047) of Southern Medical University. The animal experiments were approved by the Institutional Animal Care and Use Committee of Nanfang Hospital, Southern Medical University (Application Number: NFYY‐2020‐0736).

## Supporting information



Supporting Information

## Data Availability

The data that support the findings of this study are available in this paper and the supporting information of this article.
