# Supplementary material for: Kibra knockdown inhibits the aberrant Hippo pathway, suppresses renal cyst formation and ameliorates renal fibrosis in nphp1 KO mice
Source: Clin Transl Med. 2025 Feb 24;15(3):e70245. doi: 10.1002/ctm2.70245 (PMC11850762; doi:10.1002/ctm2.70245)
Supplement: Supplementary file 1 — Supporting Information [file CTM2-15-e70245-s001.pdf]

SUPPLEMENTAL TABLE OF CONTENTS:

Supplementary Table 1. Primers used for qPCR.
Supplementary Table 2. Primary antibodies and secondary antibodies.
Supplementary Figure 1. The statistics of the expression of the non-phosphorylated forms of the core Hippo pathway proteins after *nphp1* re-expression.
Supplementary Figure 2. Nuclear/cytoplasmic fluorescence ratio of YAP.
Supplementary Figure 3. The statistics of the expression of the phosphorylated forms of the core Hippo pathway proteins after *Kibra* knockdown in the kidney of mice.
Supplementary Dataset 1. Uncut Western Blots

Supplemental Table 1. Primers used for qPCR.

Gene	Primer	Sequence
nphp1	Forward	AGCAGGAGGGGAAGAAGT
	Reverse	TGGAAGAGTGTGGAAGGC
MST1	Forward	CTCAACTCCTACAGCACCCGTTTG
	Reverse	TCTCTTCATCATCCTGGTCCACCTC
MST2	Forward	CAGCGCCCAAGAGTAAGCTA
	Reverse	CGCCGCAGTACTCCATAACA
LATS1	Forward	CACCGAAATTTGGGACGCAT
	Reverse	TGCACACTCCCTGGTTTCAA
LATS2	Forward	TGCTCCTCCGAAAAGGATACAC
	Reverse	GAAGTCAATGGCCCCGAAGA
YAP	Forward	CAGCAGAACCGTTTCCCAGACTAC
	Reverse	GACTTGGCATCAACTCCTCTCCTTC
TAZ	Forward	CACCGTCTCCAATCACCAGT
	Reverse	TGAAGAAGCGGGAGTGTAGC
Kibra	Forward	GGACCCCAAAGCCAGTATGT
	Reverse	CTGTCTGAAGCTCACCCTTGT
NF2	Forward	TGGGAATGGCGGTGAAGGAG
	Reverse	CAGTGGGAGGTAGGAAGCAGTAG
FRMD6	Forward	TTGTGGTCACTCCCTCATGC
	Reverse	CACAAGGCATCATAGCACGC

Supplementary Table 2. Primary antibodies and secondary antibodies.

Primary antibodies	Source
anti-NPHP1	Sab2104055, Sigma-Aldrich
anti-Kibra	ab216508, Abcam
anti-MST1	ab51134, Abcam
anti-MST2	ab52641, Abcam
anti-phospho-MST1 (Thr183)/MST2 (Thr180)	49332, CST
anti-LATS1	3477, CST
anti-LATS2	20276-1-AP, Proteintech
anti-phospho-LATS1 (Ser909)	9157, CST
anti-YAP	M027159F, Abmart
anti-phospho-YAP (S127)	T55743F, Abmart
anti-TAZ	66500-1-Ig, Proteintech
anti-phospho-TAZ (Ser89)	TA4315S, Abmart

Secondary antibodies	Source
Goat Anti-Mouse HRP	FDM007, FUDE
Goat Anti-Rabbit HRP	FDR007, FUDE

Supplementary Figure 1. The statistics of the expression of the non-phosphorylated forms of the core Hippo pathway proteins after *nphp1* re-expression.

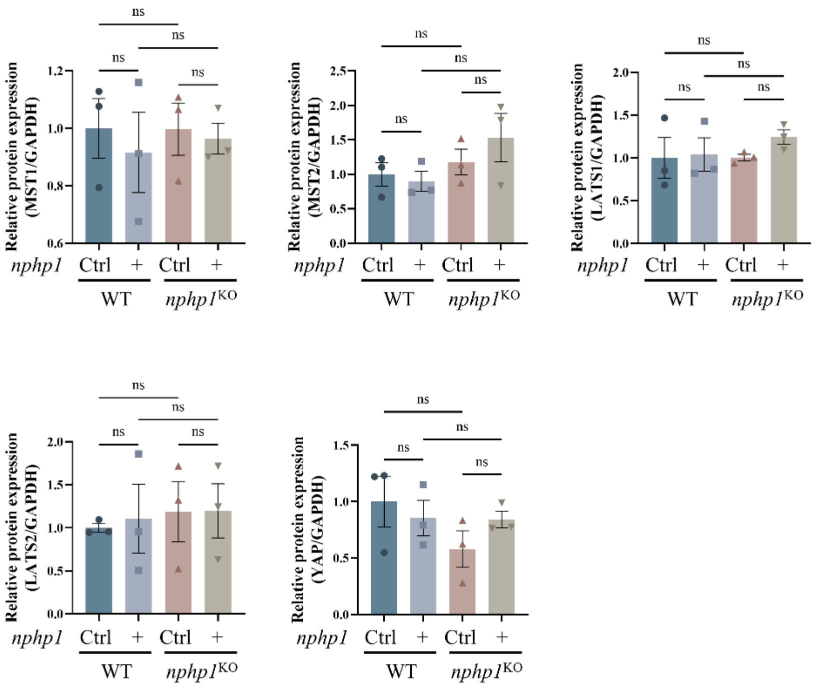

Supplementary Figure 2. Nuclear/cytoplasmic fluorescence ratio of YAP.

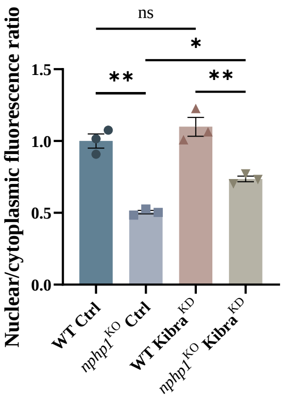

Supplementary Figure 3. The statistics of the expression of the phosphorylated forms of the core Hippo pathway proteins after *Kibra* knockdown in the kidney of mice.

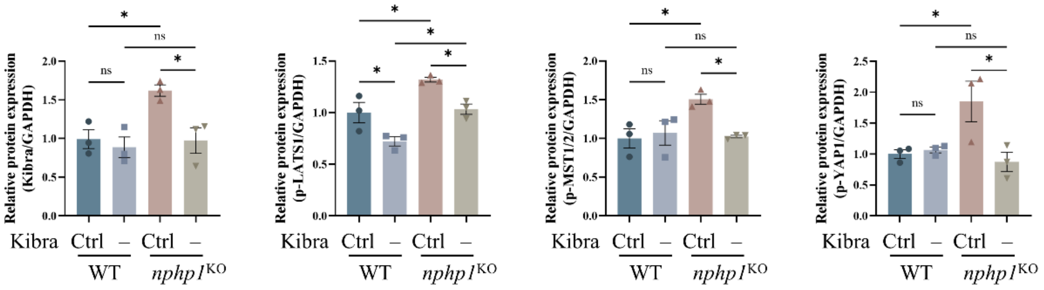

n = 3 mice/group. The data are presented as mean ± SEMs. * $p < 0.05$; ns, not significant. One-way ANOVA.

Supplementary Dataset 1. Uncut Western Blots

Figure 1B

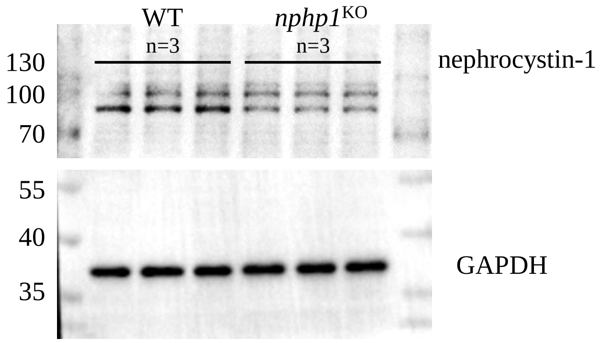

Figure 1D

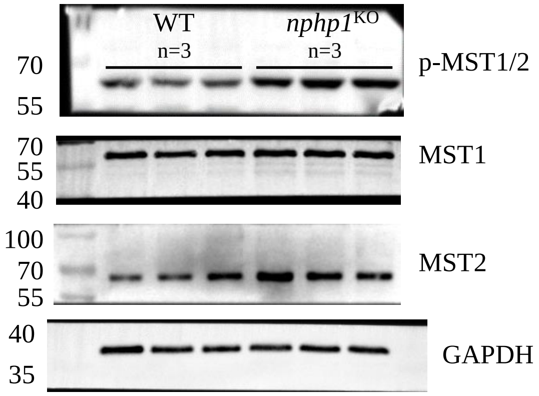

Figure 1E

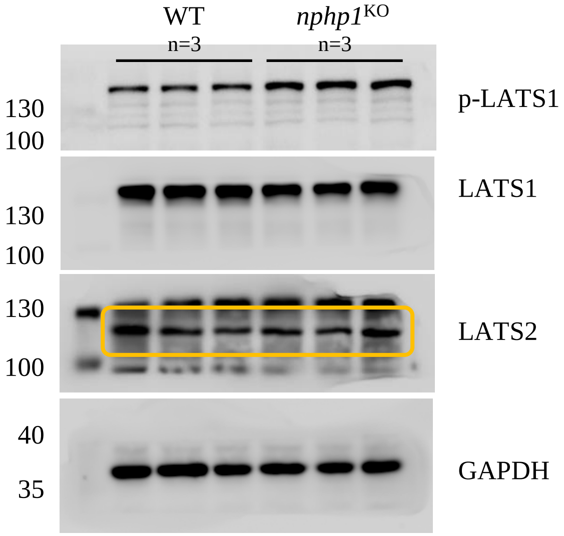

Figure 1F

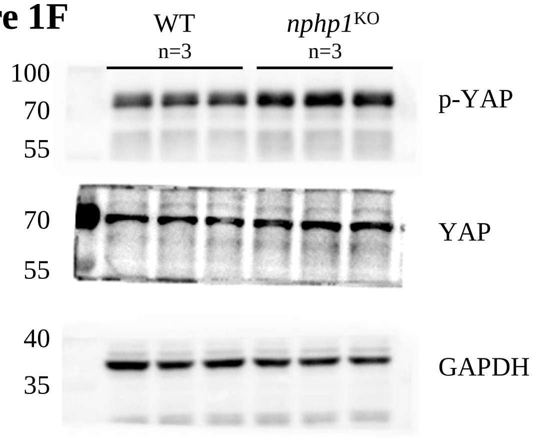

Figure 1G

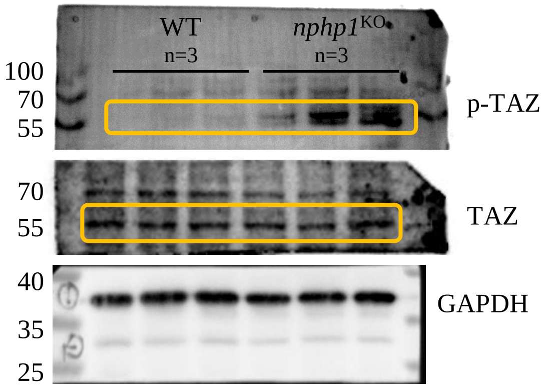

Figure 1I

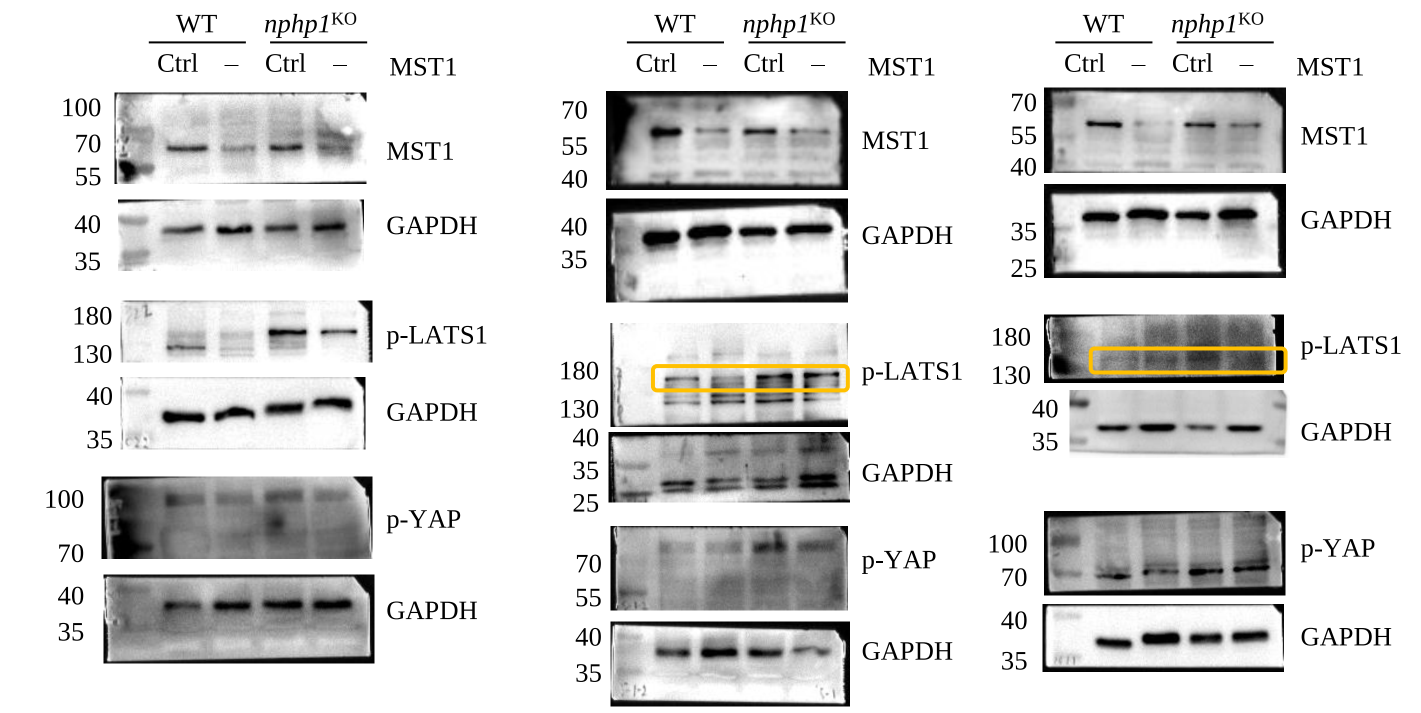

n=3

Figure 1K

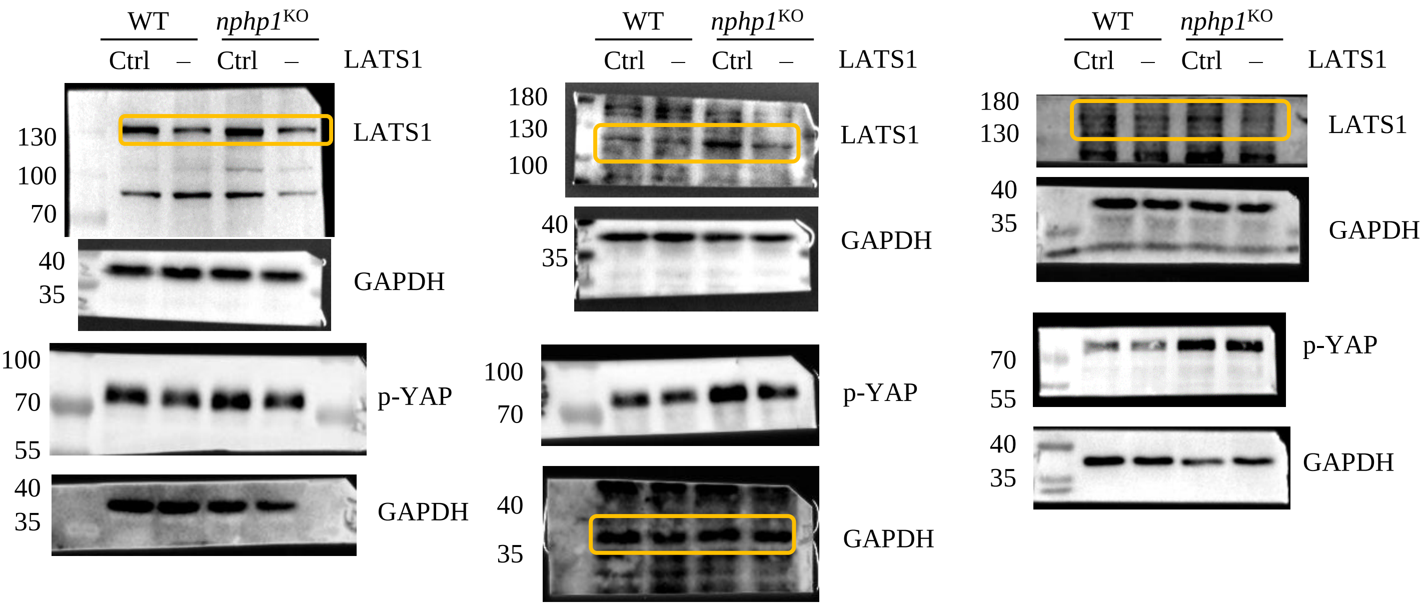

n=3

Figure 2A

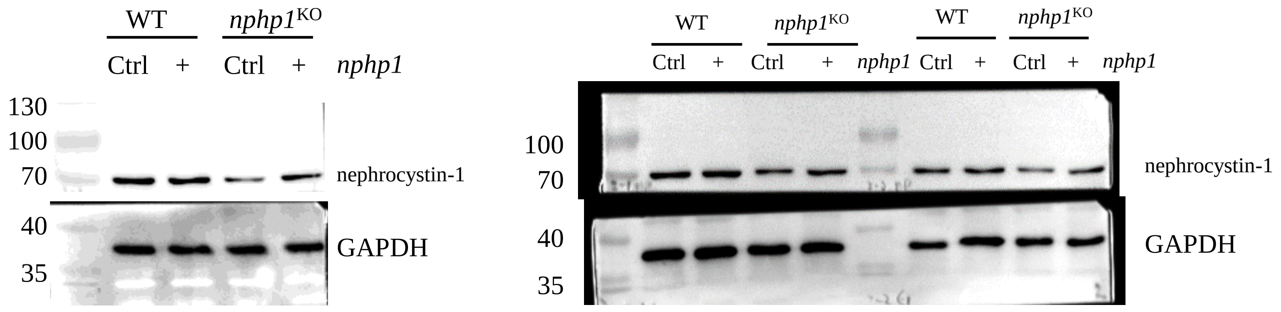

n=3

Figure 2C

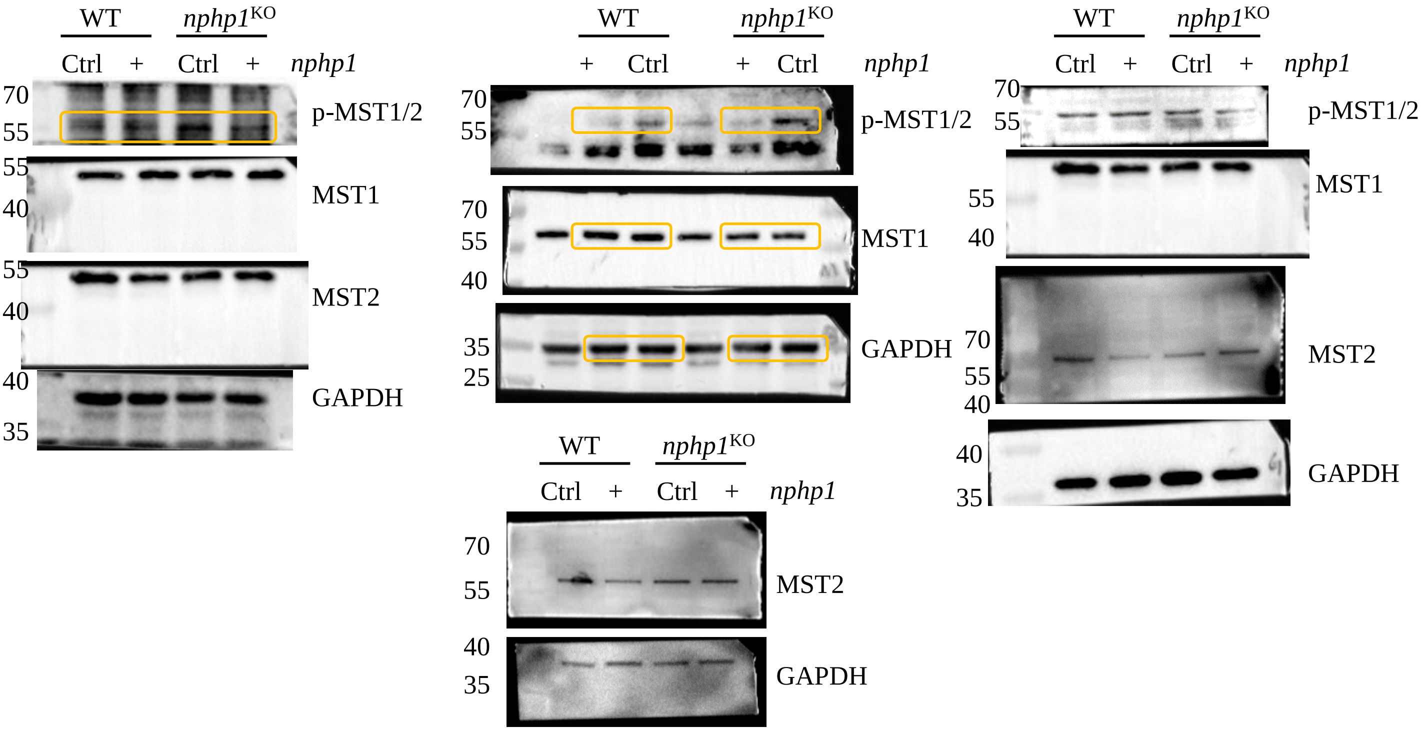

n=3

Figure 2D

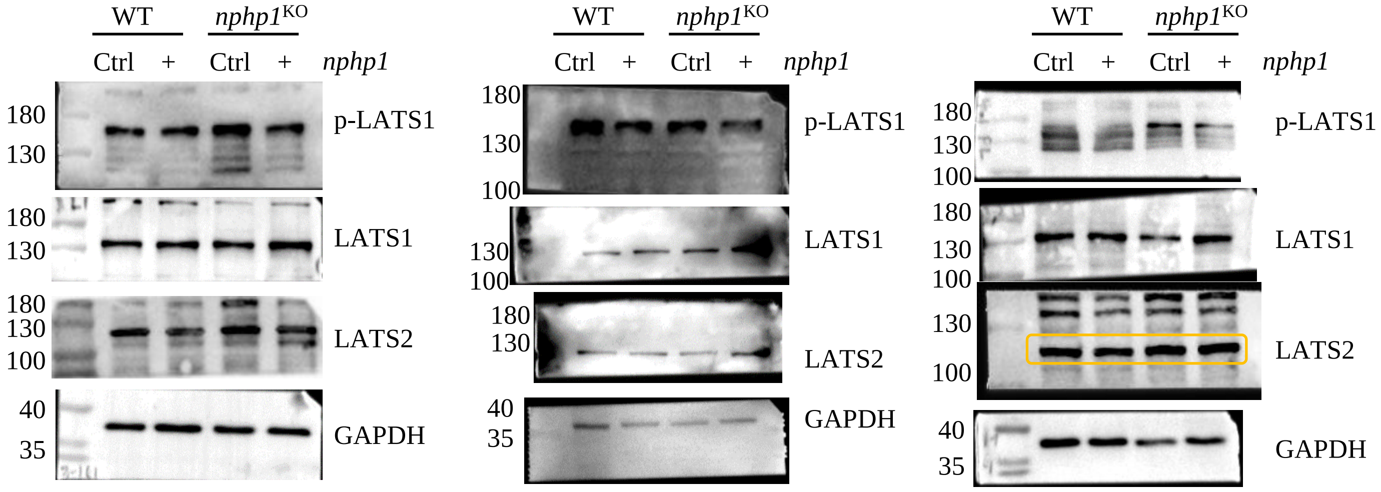

n=3

Figure 2E

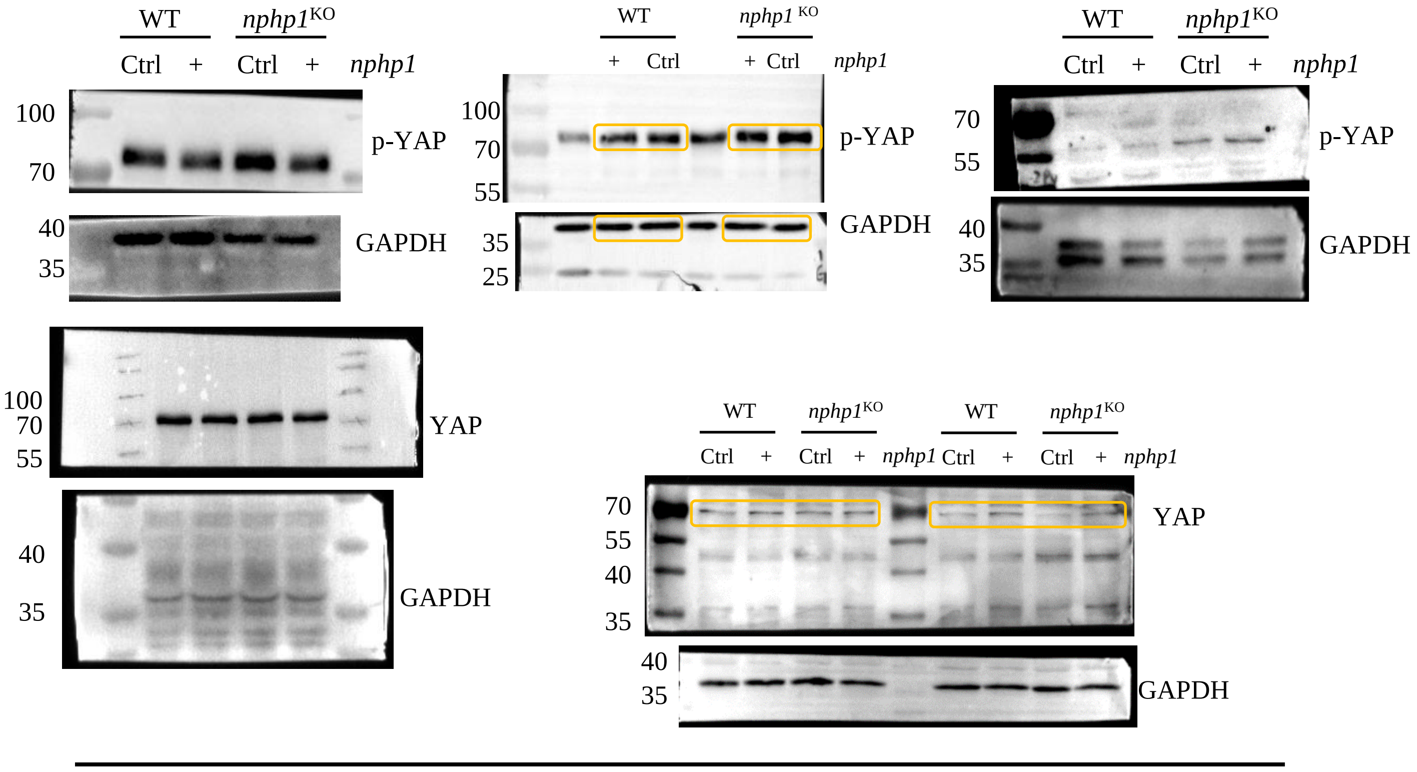

WT
nphp1KO
Ctrl + Ctrl + nphp1
100
70
p-YAP
40
35
GAPDH
WT
nphp1 KO
+ Ctrl + Ctrl nphp1
100
70
55
p-YAP
35
25
GAPDH
WT
nphp1KO
Ctrl + Ctrl + nphp1
70
55
p-YAP
40
35
GAPDH
100
70
55
YAP
40
35
GAPDH
WT
nphp1KO
WT
nphp1KO
Ctrl + Ctrl + nphp1 Ctrl + Ctrl + nphp1
70
55
40
35
YAP
40
35
GAPDH

n=3

Figure 3A

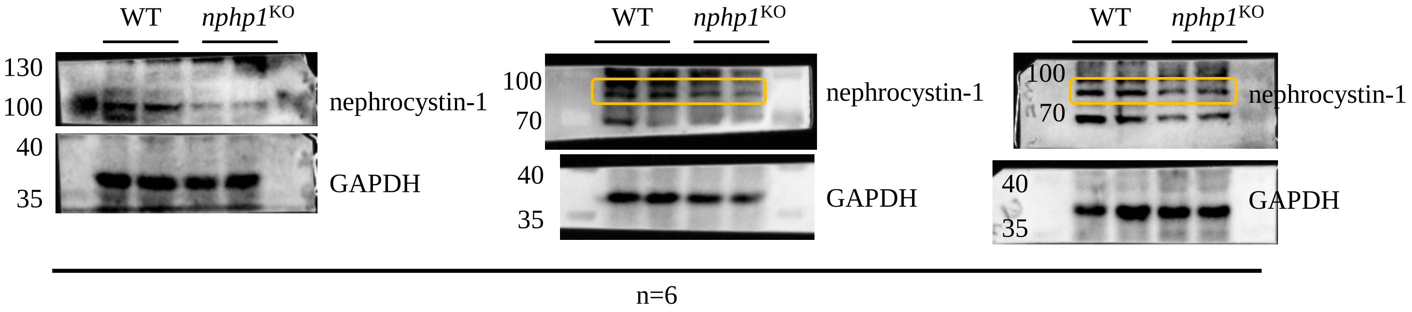

Figure 3C

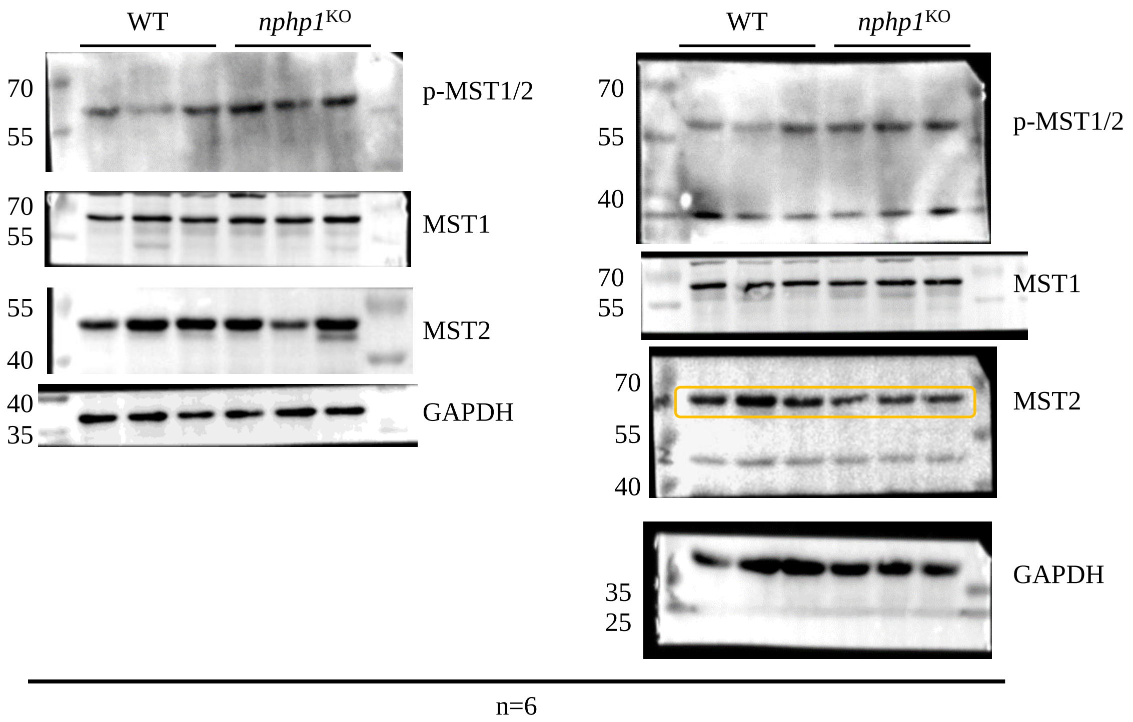

Figure 3D

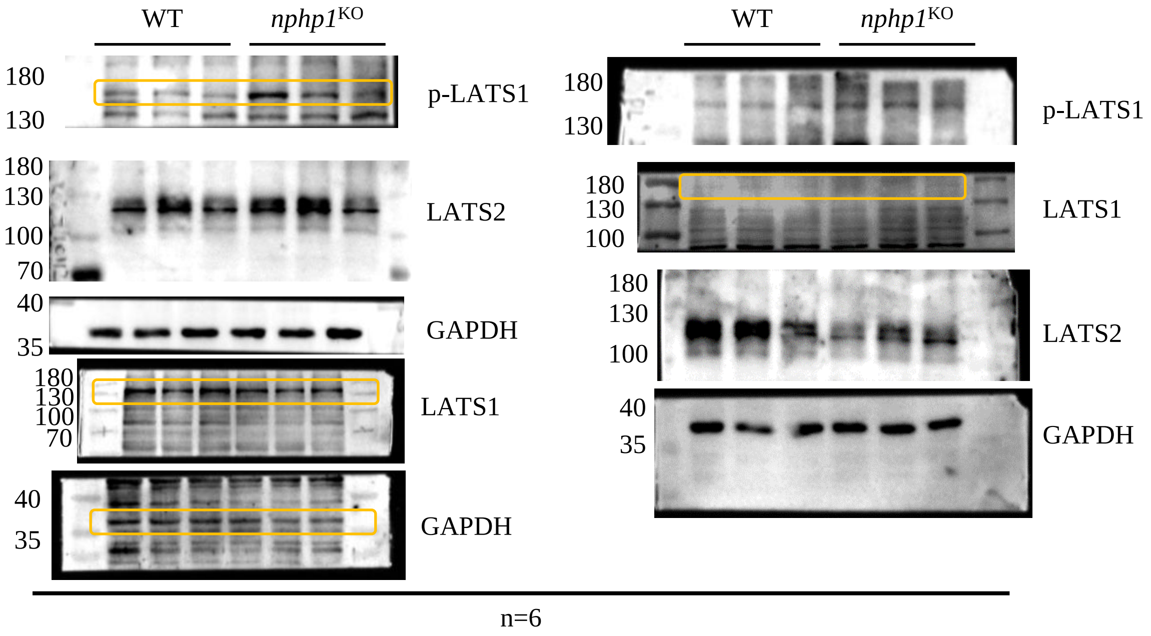

Figure 3E

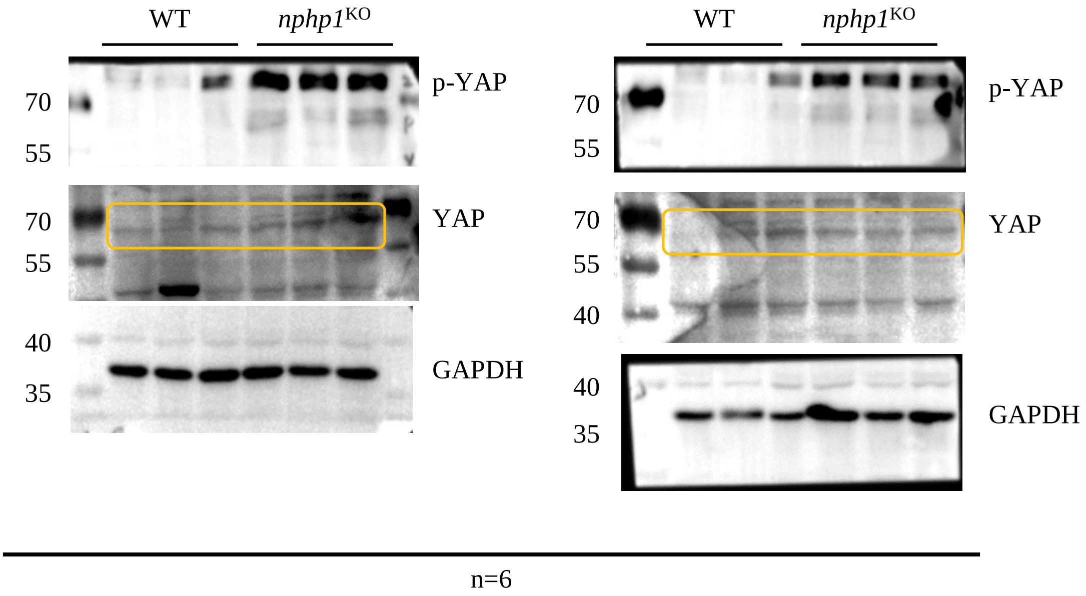
WT
nphp1KO
p-YAP
YAP
GAPDH
70
55
70
55
40
35
WT
nphp1KO
p-YAP
YAP
GAPDH
70
55
70
55
40
40
35
n=6

Figure 4B

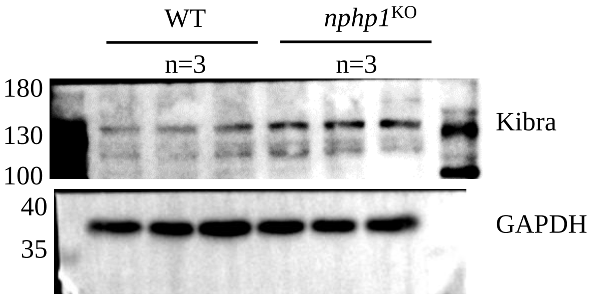

Figure 4D

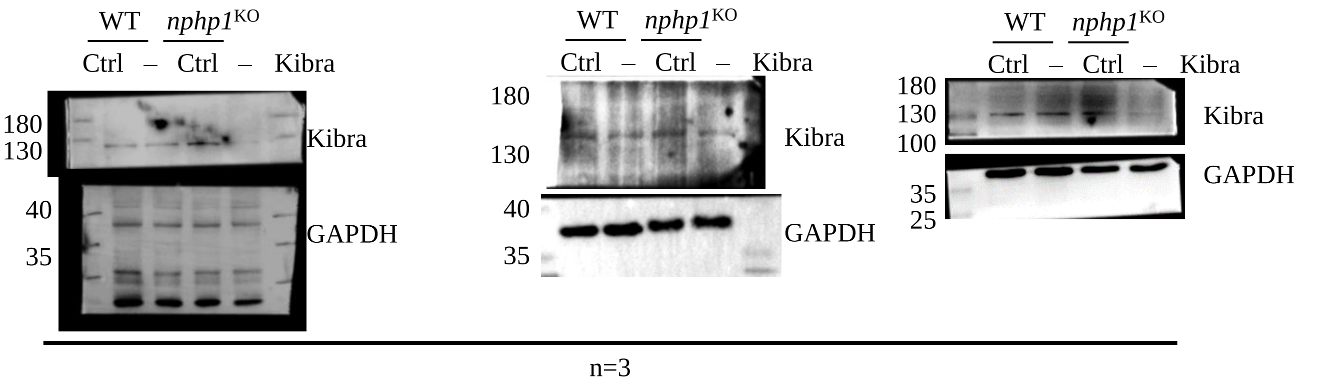

Figure 4E

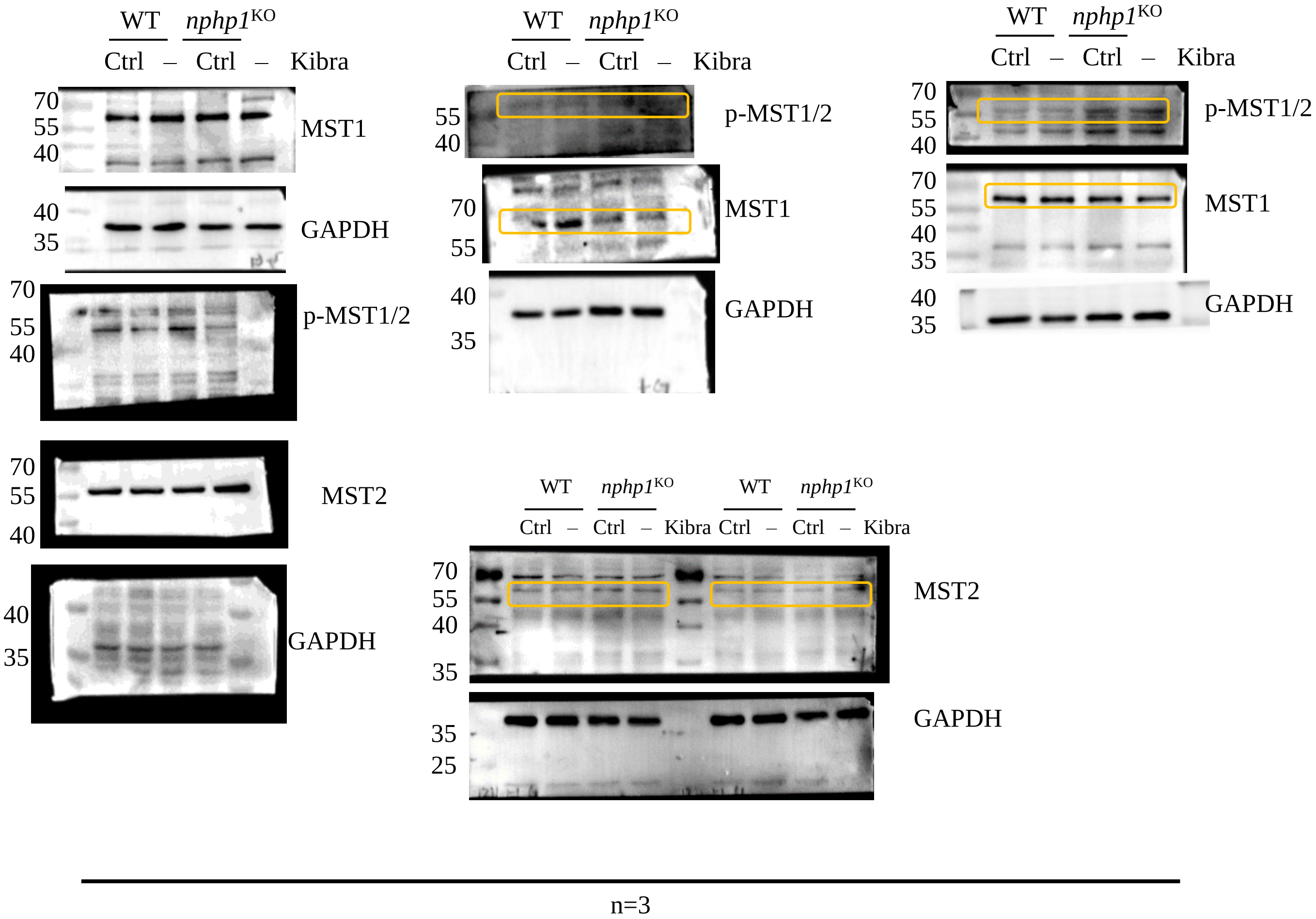

Figure 4F

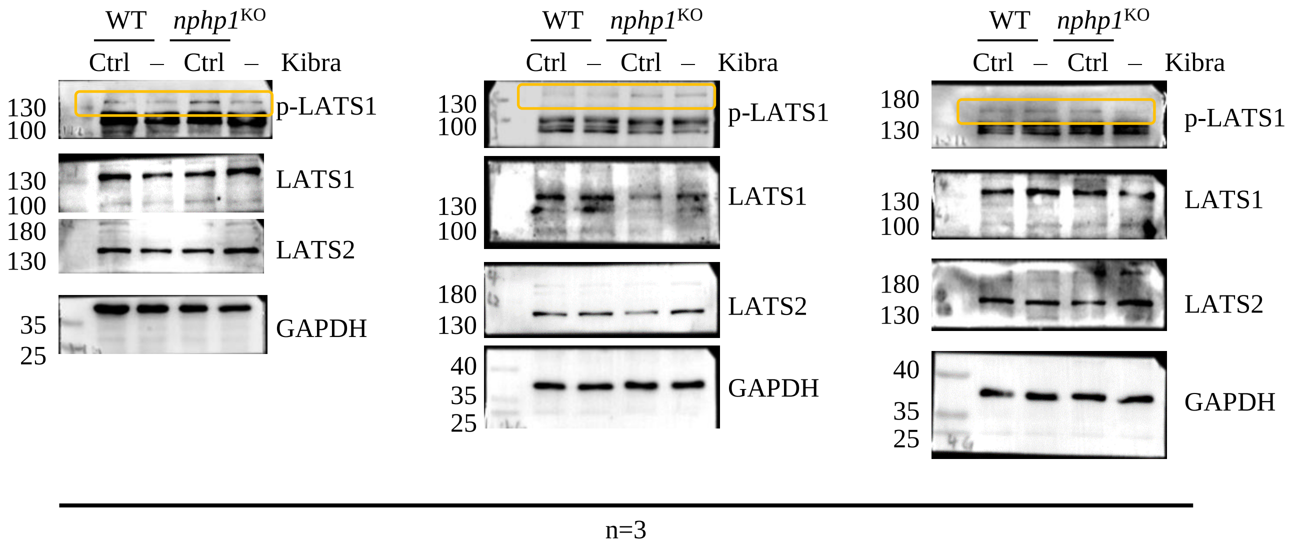

Figure 4G

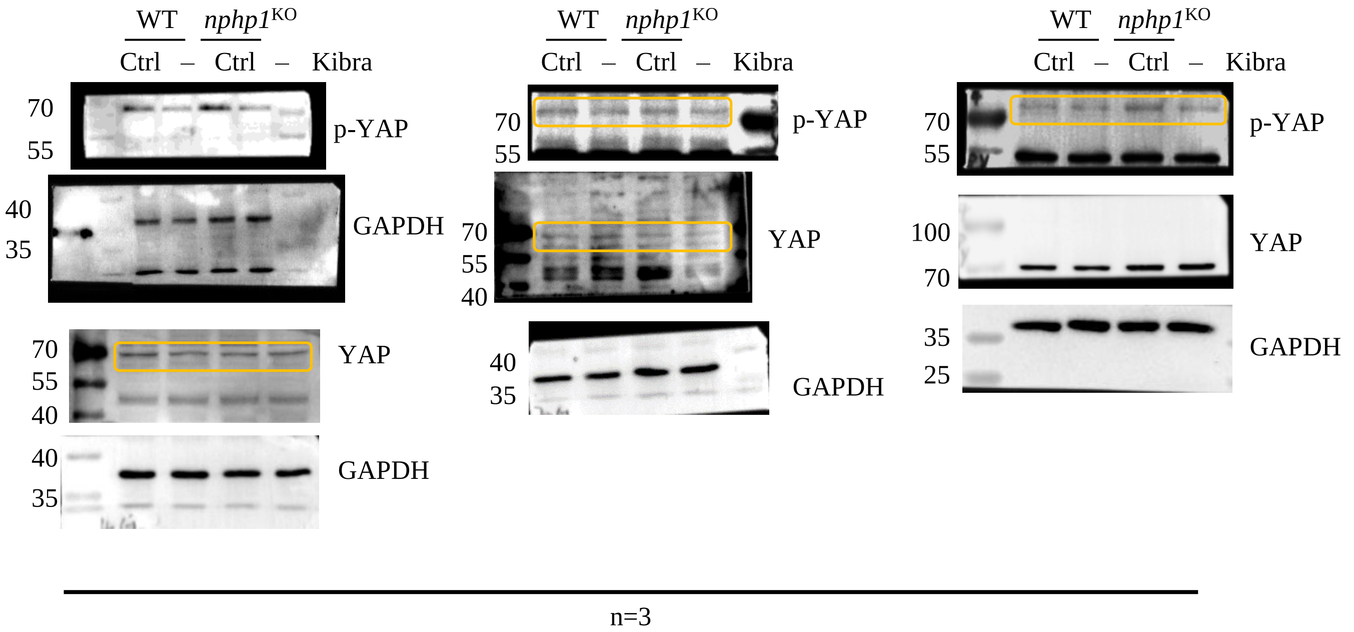

Figure 5D

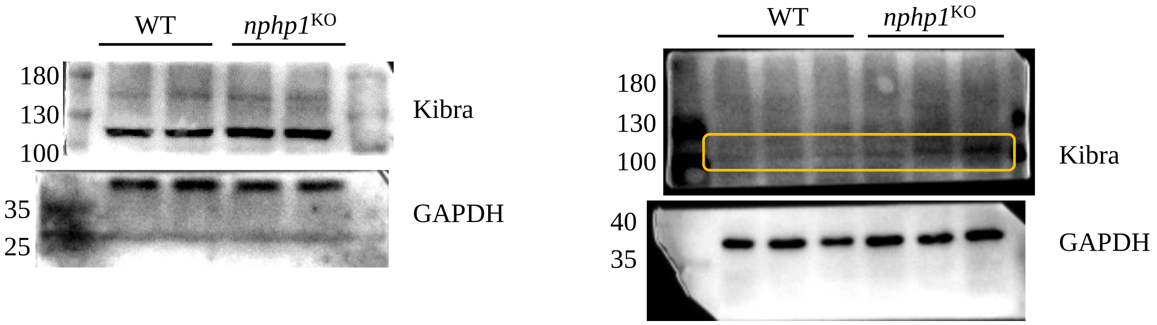
WT
nphp1KO
180
130
100
Kibra
35
25
GAPDH
WT
nphp1KO
180
130
100
Kibra
40
35
GAPDH

n=5

Figure 5F

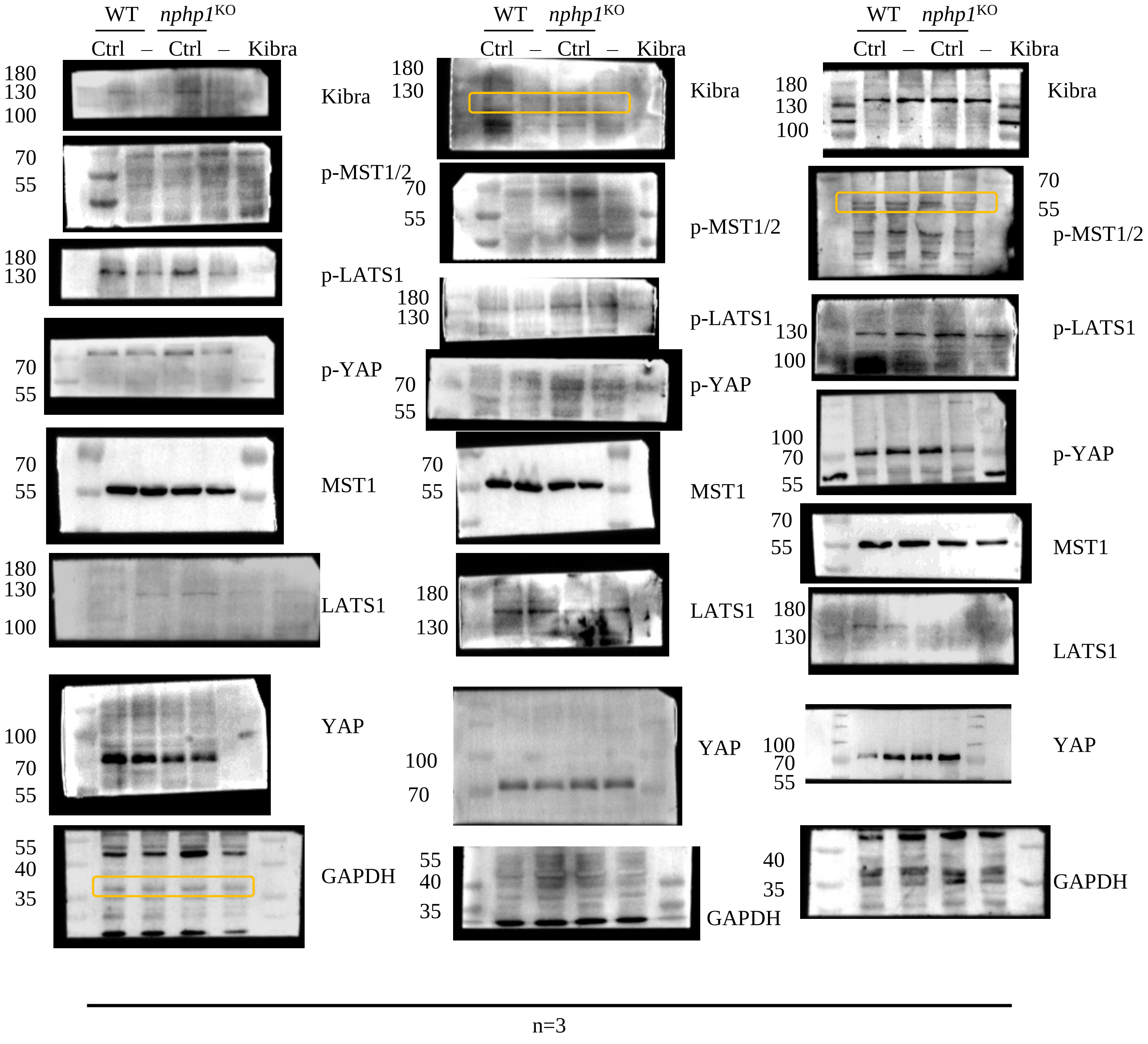
WT
nphp1KO
Ctrl
–
Ctrl
–
Kibra
Kibra
p-MST1/2
p-LATS1
p-YAP
MST1
LATS1
YAP
GAPDH
180
130
100
70
55
40
35
n=3
